# Dynamic graphs, community detection, and Riemannian geometry

**DOI:** 10.1007/s41109-018-0059-2

**Published:** 2018-03-29

**Authors:** Craig Bakker, Mahantesh Halappanavar, Arun Visweswara Sathanur

**Affiliations:** Pacific Northwest National Laboratory, 902 Battelle Boulevard, Richland, 99352 WA United States

**Keywords:** Community detection, Dynamic graphs, Riemannian geometry

## Abstract

A community is a subset of a wider network where the members of that subset are more strongly connected to each other than they are to the rest of the network. In this paper, we consider the problem of identifying and tracking communities in graphs that change over time – dynamic community detection – and present a framework based on Riemannian geometry to aid in this task. Our framework currently supports several important operations such as interpolating between and averaging over graph snapshots. We compare these Riemannian methods with entry-wise linear interpolation and find that the Riemannian methods are generally better suited to dynamic community detection. Next steps with the Riemannian framework include producing a Riemannian least-squares regression method for working with noisy data and developing support methods, such as spectral sparsification, to improve the scalability of our current methods.

**Electronic supplementary material:**

The online version of this article (10.1007/s41109-018-0059-2) contains supplementary material, which is available to authorized users.

## Introduction

### Graphs and dynamic community detection

Community detection is an important activity in graph analytics with applications in numerous scientific and technological domains (Girvan and Newman 2002). Given a graph *G*=(*V*,*E*) with weight function *w*:*E*→ℜ^+^, the goal of community detection (or graph clustering) is to partition the vertex set *V* into an arbitrary number of disjoint subsets of *V* called communities (or clusters) such that the vertices within a community are tightly connected with each other but sparsely connected with the rest of the graph. Clustering on *G* can be represented as *C*(*G*), which is a unique mapping of each vertex to a community. We restrict our work here to undirected, unweighted graphs and to the disjoint partitioning of vertices into communities. For a detailed treatment of this topic, the reader is referred to the work by Fortunato (2010).

The relationships between entities in domains such as sociology, finance, cybersecurity and biology are most naturally modeled with the use of graphs. The inherently dynamic nature of such data (Fenn et al. 2012) leads to dynamic graph representations. A dynamic graph changes over time through the addition and deletion of vertices and edges. A snapshot of this graph, *G*_*n*_, consists of the vertices and edges that are active at a given time step *n*. Modifications from time *n* to *n*+1 are represented by *ΔG*_*n*_. Clustering can be performed at each time step, *C*(*G*_*n*_), and as the graph evolves, so do its communities. Temporal communities can undergo several different transitions: *growth* via addition of new vertices, *contraction* via deletion of vertices, *merging* of two or more communities, *splitting* of a community into two or more communities, *birth* and *death* of a community, and *resurgence* or reappearance of a community after a period of time. Efficiently detecting these transitions is a challenging problem.

The problem of dynamic community detection has received significant interest in the academic literature (Cazabet and Amblard 2014). Current approaches for dynamic community detection broadly fall under two headings: incremental community detection and global community detection. The approaches in the first category focus on the systematic propagation of communities through time, whereas the approaches in the second category attempt to simultaneously optimize for multiple metrics on several snapshots of data. Stability of computation and accuracy of results are the fundamental limitations of the incremental approaches, while memory (space) and computational requirements are the main limitations of the global approaches (Cazabet and Amblard 2014). Incremental approaches are fundamentally combinatorial in nature (Tantipathananandh and Berger-Wolf 2011; Nguyen et al. 2014) and involve methods to track communities through time. The stochastic nature of these algorithms makes these methods unstable leading to inaccurate results. Mucha et al. (2010) build on the seminal work of Lambiotte et al. (2014) for community detection in dynamic multiplex networks by specializing null models in terms of stability under Laplacian dynamics.

### Motivation for a Riemannian framework

There is a well-developed suite of methods for community detection in static graphs, but it is not always clear how to extend those methods to dynamic graphs in a way that captures the time-varying nature of those graphs’ communities. The challenge is to develop methods that vary continuously in time, like the graphs themselves, between snapshots. Moreover, if existing methods are extended through time, it will be beneficial to do so in a way that provides new insight or analytical tools as well. With that in mind, we propose a Riemannian geometry approach that views dynamic graphs (and thus dynamic communities) through the lens of Laplacian dynamics on a matrix manifold. Riemannian geometry provides ways of calculating quantities such as distances between Laplacians and trajectory speeds on the matrix manifold. As such, it provides a clear and consistent way of representing graph dynamics. This framework is also modular with respect to existing static community detection methods.

### Contributions

In this paper, we provide the background theory needed to describe dynamic graphs in terms of Laplacian dynamics on matrix manifolds. The primary contribution of this paper is to bring existing theory to bear on a new application area – dynamic community detection. We use Riemannian geometry to interpolate between snapshots of dynamic graphs (using geodesics) and to calculate averages of those snapshots; we explicitly show the formulae for performing these calculations. The interpolated and average graphs are then amenable to existing static community detection methods. This allows us to use a consistent approach to track community behaviour both between snapshots, via interpolation, and across snapshots, via averaging.

Simply transferring previously derived formulae would not allow us to consider disconnected graphs, however, so our contributions also include a way of transforming disconnected graphs so that they are amenable to the matrix manifold tools. Using both synthetic and experimental graph data, we experimentally evaluate two different kinds of geodesics. We identify their strengths, as compared with entry-wise linear interpolation, and also discuss their weaknesses. Finally, we derive interpolation and extrapolation error bounds for both geodesics (shown in the Appendix) and identify promising avenues of future research in this area.

Our framework enables more accurate prediction of community transitions by building interpolated graphs between snapshots, global community detection through data aggregation, and prediction of future behaviour through extrapolation from given snapshots. We describe the basics of our framework in the “Riemannian geometry and dynamic graphs” section, show how it can be applied to dynamic clustering in “A Riemannian framework for dynamic community detection” section, and compare the Riemannian methods with an entry-wise linear approach on synthetic and real network data in the “Computational experiments” section.

The novelty of our approach arises primarily from the application of Riemannian geometry to dynamic graphs. When combined with existing spectral methods, this also provides a new interpretation of community splitting and merging as bifurcations in a gradient flow dynamical system (see the “Dynamic spectral clustering” section). To the best of our knowledge, the Riemannian framework presented in this paper is the first of its kind; it is our intent that the research community build from and extend this work to enable features of dynamic community detection not currently considered here.

## Riemannian geometry and dynamic graphs

### Riemannian geometry and matrix manifolds

Differential geometry deals with mathematics on manifolds; manifolds are spaces that are locally Euclidean (i.e., flat), but generally non-Euclidean globally (Boothby 1986). A Riemannian manifold is a type of manifold that has a metric associated with each point on the manifold. The traditional methods for calculating angles and distances in flat spaces have to be modified on manifolds to account for manifold curvature, and the metric is an integral part of those modifications on Riemannian manifolds.

A key part of Riemannian geometry, for the purposes of this paper, is the geodesic. Geodesics are the equivalent of straight lines in curved spaces. A geodesic is (locally) the shortest path between two points. Great circles on a sphere are examples of geodesics on a curved manifold. Consider a flight from Vancouver, Canada to London, England: the two cities are at similar latitudes, so on a Mercator projection map, the shortest flight would seem to be a straight West-to-East trajectory. In reality, however, flights between the two cities traverse the Pole because that is a shorter route – it is the great circle route. The discrepancy is due to the curvature of the Earth, which is distorted on a flat map. From another perspective, a geodesic is the path that a particle on a manifold would take if it were not subject to external forcing; a geodesic with constant speed has zero acceleration.

Riemannian geometry can be applied to matrix manifolds. The Grassman and Stiefel manifolds are perhaps the most frequently encountered matrix manifolds in differential geometry because they have closed-form solutions for quantities such as geodesics (Absil et al. 2007). Pennec et al. (2006) developed a metric for the manifold of symmetric positive-definite matrices with corresponding expressions for distances, geodesics, and tangent vector inner products *in closed form*. These formulae are valuable because even when there is a well-defined metric on a manifold, distances and geodesics between points do not usually have closed-form expressions. Such quantities have to be solved for numerically. Working on this matrix manifold, when appropriate, can be useful: matrix symmetry provides a reduction in effective dimension, and properties such as symmetry and positive-definiteness are automatically preserved.

Bonnabel and Sepulchre (2009) extended this framework to include symmetric positive-semidefinite matrices. The extension essentially worked by decomposing a positive-semidefinite matrix into a nullspace component (a Grassman manifold) and a positive-definite component, which could then use the existing metric.

### Graph Laplacians and Riemannian geometry

Researchers have previously used non-Euclidean geometries to investigate graphs (Krioukov et al. 2009, 2010). That work has then been applied to large-scale networks such as the internet (Boguná et al. 2010). The approach described in this paper differs in a subtle but meaningful way. In those papers, the mappings used treat graph nodes as points in a hyperbolic space. Our present work, however, treats the entire graph as a single point in a non-Euclidean space.

The work of Bonnabel and Sepulchre (2009) combined with that of Pennec et al. (2006) enables us to consider graph Laplacians as points on a manifold of positive-semidefinite matrices. Each graph is a point, and thus a time-indexed sequence of graphs forms a trajectory on the manifold. This, in turn, means that we can calculate quantities such as trajectory velocities, distances between graphs (represented by manifold distances between their respective points), and relevant geodesics.

Given that we are interested in dynamic community detection, the Laplacian is a natural object to work with. The Laplacian uniquely defines a graph (up to self-loops), and there is already a known connection between the Laplacian spectrum and community structure (Newman 2010). Previous work in dynamic community detection (e.g., Mucha et al. (2010)) has also worked with the Laplacian. Graph Laplacians have a certain structure that make them amenable to the Riemannian geometry techniques presented here as well: Laplacians are symmetric (for undirected graphs) and positive-semidefinite. Adjacency matrices, for example, are generally indefinite and thus would not be suitable for use with the matrix manifolds described here.

We chose to work with the combinatorial Laplacian, *L*=*D*−*A*, because it has a constant nullspace for connected graphs (Newman 2010). This constant nullspace makes the geometric calculations much simpler than they would be otherwise. It is possible to use other Laplacians, such as the normalized Laplacian. If these Laplacians do not have constant nullspaces, though, the interpolation involves extra calculations (detailed by Bonnabel and Sepulchre (2009)). Assuming no self-loops, the combinatorial Laplacian also has the virtue of being easy to convert into an adjacency matrix. That being said, as long as a Laplacian is symmetric positive-semidefinite and has a constant nullspace dimension (for connected graphs), it is possible to calculate geodesic interpolations for that Laplacian.

There are two other relevant considerations we wish to address here. Firstly, the Laplacians of unweighted graphs constitute a discrete (and therefore sparse) subset of the matrix manifold. As such, any *continuous* trajectory will contain weighted graphs. Secondly, directed graphs do not have symmetric Laplacians, and thus they cannot be considered within this framework without symmetrizing them somehow (e.g., by ignoring the directionality of edges). For the purpose of community detection, though, edge direction may not be important.

## A Riemannian framework for dynamic community detection

There are two primary components to our framework. The first involves modelling and analyzing the dynamic behaviour of the graph prior to any community detection. For this, we show how to calculate an average graph from a collection of snapshots (for use in a time-averaged community detection) and how to interpolate between time-indexed graph snapshots (for seeing how the graph evolves over time). In the Appendix, we derive and analyze bounds on the interpolation error in terms of distance on the manifold.

The second component consists of applying community detection methods to the dynamic graph. In this paper, we will focus on spectral methods, because they have convenient properties under continuous Laplacian dynamics, and the Louvain method (Blondel et al. 2008), because of its computational speed and ability to handle disconnected graphs. However, the Riemannian geometry methods do not require using any one particular community detection method.

### Graph interpolation and averaging

We begin with interpolation between two snapshots. It is possible to do this using an entry-wise linear approach, *L*(*t*)=(1−*t*)*L*_*A*_+*tL*_*B*_, but there are good reasons not to use this approach.

Firstly, the Laplacians for a given dynamic graph all exist on a matrix manifold. For the trajectory *L*(*t*) on that manifold, though, the trajectory speed is not constant, the trajectory direction is not constant, and it is not the shortest path from *L*_*A*_ to *L*_*B*_. It is precisely analogous to the Mercator projection map example given earlier – moving at a constant velocity (i.e., constant speed and direction) on the map would not correspond to moving at a constant velocity on the earth because of the earth’s curvature. Experimentally, we have observed that the linear interpolation begins and ends its trajectory moving very quickly while the bulk of its trajectory moves relatively slowly. The difference between maximum and minimum velocities can be orders of magnitude, depending on the size of the graph and the distance between the two graphs being interpolated.

Secondly, in connected graphs, the product of the Laplacian’s non-zero eigenvalues (i.e., the determinant of the positive-definite component) is concave along the linearly interpolated trajectory. If the two points are far enough apart, this product will go through a maximum between the two points. This maximum can, again, be orders of magnitude greater than the product at either endpoint; like the trajectory velocity, this variation will depend on the size of the graphs in question and their distance apart. The geodesic interpolation, however, provides a linear variation in the product of the eigenvalues. Pennec et al. (2006) comment on this in more detail. For a graph, this product relates directly, by Kirchoff’s matrix tree theorem, to the number of spanning trees in the graph (Harris et al. 2008). In other words, the linear interpolation increases the overall connectivity of the graph between snapshots.

Finally, the linear interpolation cannot always be used for extrapolation. All of the interpolated Laplacians are positive-semidefinite, but it is easy to provide examples where the extrapolation quickly becomes indefinite.

Instead, we propose using geodesic interpolation. A geodesic interpolation trajectory has a constant velocity, produces an eigenvalue product that varies linearly between endpoints that are connected graphs, and can be extrapolated indefinitely without leaving the manifold of positive-semidefinite manifolds (with constant nullspace dimension). Following Bonnabel and Sepulchre (2009), we show how to calculate this geodesic between two snapshots of a given dynamic graph.

Consider the Laplacian *L* at a point. It can be represented with its eigendecomposition:

1 $$ L = \left[ \begin{array}{cc} \alpha &\xi \end{array} \right] \left[ \begin{array}{cc} D &0 \\ 0 &0 \end{array} \right] \left[ \begin{array}{c} \alpha^{T}\\ \xi^{T} \end{array} \right] = \alpha D \alpha^{T}  $$

where the columns of *α* span the range of *L*. Moreover, the nullspace, *ξ*, is always parallel to (1,1,…,1), and thus span(*α*) is constant even though *α* may not be, in general.

Consider the geodesic between *L*_*A*_ and *L*_*B*_. We can calculate the SVD of $\alpha _{B}^{T} \alpha _{A}$:

2 $$ \alpha_{B}^{T} \alpha_{A} = O_{B} \sigma_{AB} O_{A}^{T}  $$

The diagonal matrix *σ*_*AB*_ has the principal angles between the subspaces spanned by *α*_*A*_ and *α*_*B*_ as its diagonal entries. Since those subspaces are the same, *σ*_*AB*_=*I* for any two Laplacians. We then calculate *U*_*A*_=*α*_*A*_*O*_*A*_ and *U*_*B*_=*α*_*B*_*O*_*B*_. Since *σ*_*AB*_ is constant, *U*_*A*_=*U*_*B*_, and *U* is constant for all points on the geodesic; *α* and *O* are not constant, though. Furthermore, we can use the same *U* matrix for any Laplacian of a given dynamic graph without affecting our calculations, because the span of *U* is constant. We calculate *R*=*U*^*T*^*LU* for *L*_*A*_ and *L*_*B*_. The geodesic from *L*_*A*_ at *t*=0 to *L*_*B*_ at *t*=1 is then

3 $$\begin{array}{*{20}l} R (t) &= R_{A}^{\frac{1}{2}} \exp \left(t \ln R_{A}^{-\frac{1}{2}} R_{B} R_{A}^{-\frac{1}{2}} \right) R_{A}^{\frac{1}{2}}  \end{array} $$

4 $$\begin{array}{*{20}l} L(t) &= U_{A} R (t) U_{A}^{T} = U_{B} R (t) U_{B}^{T} \end{array} $$

If there are multiple time-sequenced snapshots, this method can be used to do a piecewise geodesic interpolation with *t* being shifted and scaled appropriately. Note that the constant Laplacian nullspace means that we can work solely with the *R* components of *L* and ignore the Grassman component. We can also extrapolate with this geodesic simply by continuing the trajectory for *t*>1.

If we are interested in the average behaviour of a dynamic graph, we can calculate the least-squared-distance mean (the Karcher mean) of a set of graph snapshots. To do this, we use the *R* matrices derived from the graph Laplacians as before; each graph *i* has a matrix *R*_*i*_ associated with it, and we want to determine the ‘average’ matrix *S* for *N* snapshots. We then list the sum-of-squared-distance function, the distance function itself, and the gradient of the squared distance (Pennec et al. 2006), respectively:

5 $$\begin{array}{*{20}l} f (S) &= \frac{1}{2N} \sum_{i} f^{(i)} = \frac{1}{2N} \sum_{i} d^{2} \left(S,R_{i}\right) \end{array} $$

6 $$\begin{array}{*{20}l} d^{2} \left(S,R_{i}\right) &= \left\| \ln S^{-\frac{1}{2}} R_{i} S^{-\frac{1}{2}} \right\|^{2} \end{array} $$

7 $$\begin{array}{*{20}l} \nabla_{S} f^{(i)} (S) &= - 2 \ln_{S} R_{i} \end{array} $$

We use iterated gradient descent to calculate the mean:

8 $$ S_{k+1} = S_{k}^{\frac{1}{2}} \exp \left[ \frac{1}{N} \sum_{i} \ln \left(S_{k}^{-\frac{1}{2}} R_{i} S_{k}^{-\frac{1}{2}} \right) \right] S_{k}^{\frac{1}{2}}  $$

According to Pennec et al. (2006), this usually converges quickly.

### Alternative Riemannian geometries

Riemannian geometry centers around the Riemannian metric – changing the metric entails changing properties of the manifold (such distances and geodesics). The current metric can be described as affine-invariant (Pennec et al. 2006), but it is not the only metric that could be used for the space of positive-definite matrices. We could also use a log-Euclidean metric as described by Arsigny et al. (2007). The primary reason to consider using the log-Euclidean metric instead of the affine-invariant one is computational cost: the formulae for distances and geodesics are simpler and easier to calculate for the log-Euclidean metric. Those distance and geodesic formulae are, respectively,

9 $$\begin{array}{*{20}l} d^{2} \left(S, R_{i} \right) &= \left\| \ln R_{i} - \ln S \right\|  \end{array} $$

10 $$\begin{array}{*{20}l} R (t) &= \exp \left(\left(1-t\right) \ln R_{A} + t \ln R_{B} \right)  \end{array} $$

Another computationally beneficial feature of the log-Euclidean metric is the closed-form expression that it has for calculating the mean of a set of matrices:

11 $$ S = \exp \left(\frac{1}{N} \sum_{i} \ln R_{i} \right)  $$

To utilize these formulae for interpolating between graphs, we would simply replace Eq. 6 with Eqs. 9, 3 with Eq. 10, and the iterated process in Eq. 8 with a single evaluation of Eq. 11. There are other expressions that are simpler to evaluate for the affine-invariant metric, but those quantities may not be needed, and the different invariance properties of each metric may be valuable in different circumstances.

On a practical level, the two metrics generally produce similar interpolations (Arsigny et al. 2007): the spectrum of the affine-invariant interpolations tends to be slightly more isotropic than that produces by the log-Euclidean interpolations, but both interpolate determinants linearly between interpolation points (see the “Graph interpolation and averaging” section). For the rest of this paper, we will distinguish the geodesics and means calculated with the two methods as being either affine-invariant (AI) geodesics or log-Euclidean (LE).

### Disconnected graphs

The methods described in this paper currently assume that the graph in question is connected and remains so at all points of interest. As they stand, they could potentially handle a graph with a constant number of disconnected components (which would correspond to the Laplacian nullspace having a constant dimension), but this does not significantly improve the method’s generality. In order to be widely applicable, the interpolation methods need to be able to handle changing connectivity.

We can accommodate this by using a bias term with, potentially, a thresholding procedure. For a given adjacency matrix *A*, we add to each off-diagonal entry a bias term *ε*/*n*, where *ε*≪1 and *n* is the number of vertices in the graph, to produce a biased adjacency matrix $\tilde {A}$ (which is now connected). We then construct a biased Laplacian matrix from $\tilde {A}$, perform the interpolation on the biased Laplacian and subtract *ε*/*n* from each off-diagonal entry of the adjacency matrices produced by the biased interpolation. If need be, we can then apply a threshold to the resulting adjacency matrices or round those matrices to an appropriate number of decimal places. This approach essentially replaces the Laplacian’s *λ*=0 eigenvalues with *λ*=*ε*.

Empirically, we found that this approach did not significantly change the interpolated trajectories for connected graphs while also producing reasonable results for disconnected graphs. If we consider the properties of the Riemannian metrics discussed in this paper, we can see why adding this small bias would not significantly disturb a geodesic trajectory. With these metrics, matrices with zero or infinite eigenvalues essentially exist at infinity. For matrices with finite eigenvalues greater than zero, the distances between matrices are relative and directly tied to the matrices’ spectra. For example, the distance from *λ*=10^−6^ to *λ*=10^−5^ is comparable to the distance from *λ*=1 to *λ*=10. This means that a geodesic, which is a minimum-distance path between points, will not significantly alter the part of the spectrum associated with *λ*=*ε* values unless it is absolutely necessary to do so in order to reach the destination. Moreover, adding a fully connected graph with edge weights of *ε* would not meaningfully change the community structure because of the separation of scales (presuming a very small value of *ε*).

In our computational experiments, we found that *ε*=10^−6^ provided a good balance between avoiding ill-conditioning and keeping *ε* small, but even increasing *ε* to 10^−3^ did not change the interpolation significantly. As we increased *ε*, though, we found that the geodesic interpolations approached the trajectory of the linear interpolation; at, say, *ε*=10^6^, they were almost identical. This, too, makes sense: as the eigenvalues become uniformly larger, the manifold becomes flatter, and the differences between the data points become smaller. The flatter the manifold, the closer the geodesic is to the linear interpolation. However, the geodesic interpolation is still guaranteed to remain positive definite, and the linear interpolation is not. This suggests that if the linear interpolation were more desirable in a particular application but the application also called for the use of extrapolation, then using a geodesic with a large bias term could provide the desired capabilities.

### Dynamic spectral clustering

It is possible to use spectral clustering with the first non-trivial eigenvector for community detection, but this method can be improved upon by using multiple eigenvectors (Boccaletti et al. 2006). This approach is convenient for continuous Laplacian dynamics because as long as the eigenvalues are distinct, we can expect the eigenvectors and eigenvalues to vary smoothly with smooth changes in *L*. If the eigenvalues of the eigenvectors in question are not distinct, then the eigenvectors are not uniquely defined, and if eigenvalues whose eigenvectors are being used for spectral clustering cross during the course of a trajectory, the spectral clustering may experience a discontinuous jump. Disconnected graphs can provide exactly this kind of behaviour (e.g., with multiple zero eigenvalues). Moreover, if the number of disconnected components is not constant, then it will not suffice simply to consider the first *m* non-zero eigenvalues, for the set of such eigenvalues will not be constant.

Assume that the graphs are connected, that there is an ordering of the eigenvalues of *L* such that *λ*_*i*_≤*λ*_*i*+1_, *λ*_1_=0, and that eigenvector ***ξ***_(*i*)_ is associated with *λ*_*i*_. We can then plot each of the graph nodes in ℜ^*n*^, where node *k* has coordinates given by $\left (\xi ^{k}_{(2)},\xi ^{k}_{(3)}, \ldots, \xi ^{k}_{\left (n+1\right)}\right)$, and use clustering techniques to identify communities.

**Fig. 1:**
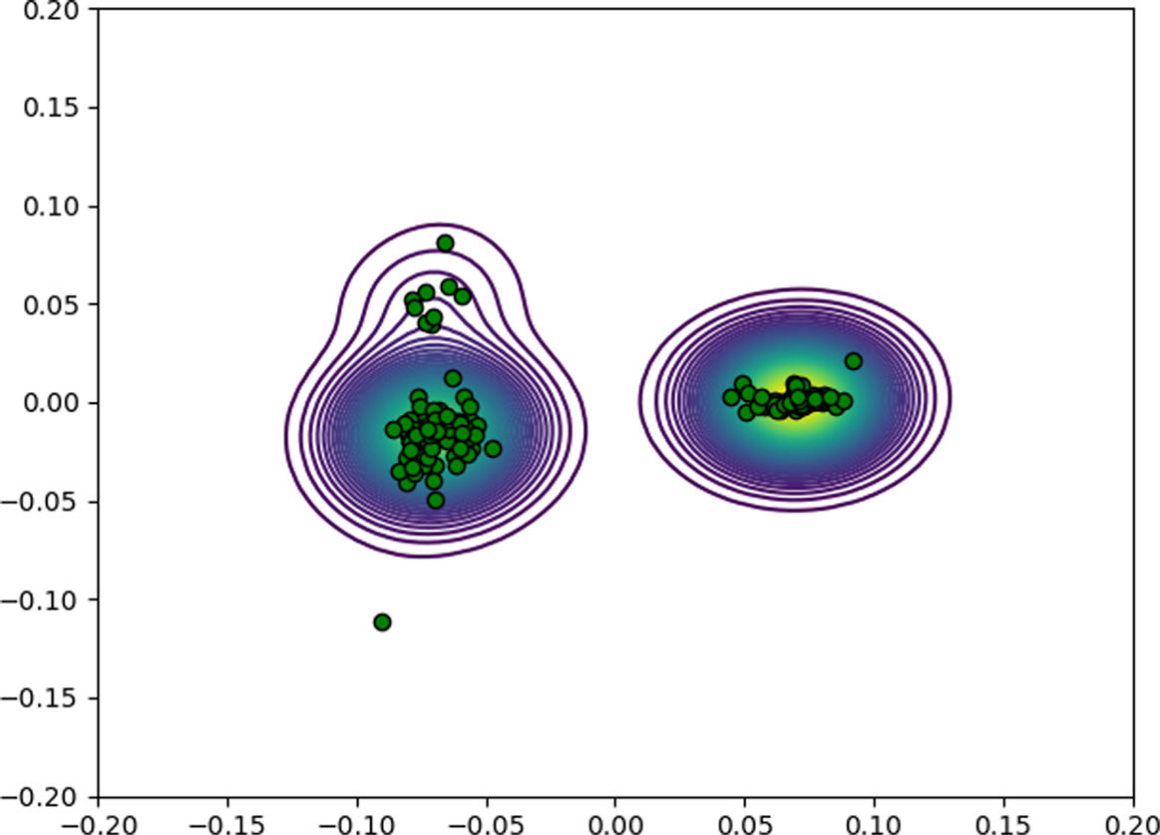
2-D spectral plot of graph nodes. The graph nodes are plotted as points, the contours show the magnitude of the density function, and the horizontal and vertical axes correspond to the ***ξ***_(2)_ and ***ξ***_(3)_ components, respectively. This particular plot shows two distinct communities with one node at approximately (-0.09,-0.11) that does not belong very strongly to either community and a cluster of points around (-0.07,0.05) that seems close to forming its own community

One way of identifying and tracking communities is through defining a kernel for the nodes. Summing over all of the nodes then produces a density function. The maxima of that density function correspond to cluster centroids, and the separatrices between maxima define community boundaries in the (reduced) eigenspace. With a symmetric Gaussian kernel, this density function would be

12 $$\begin{array}{*{20}l} f \left(\mathbf{x}\right) &= \sum \limits_{k} N \left(\mathbf{x} - \mathbf{y}_{(k)}, \sigma \right) \end{array} $$

13 $$\begin{array}{*{20}l} N \left(\mathbf{x} - \mathbf{y}_{(k)}, \sigma \right) &= \exp \left(-\frac{\left\|\mathbf{x} - \mathbf{y}_{(k)}\right\|^{2}}{2 \sigma^{2}} \right) \end{array} $$

where $y^{l}_{(k)} = \xi ^{k}_{\left (l-1\right)}$. Other kernels could be used, but this provides an easily differentiable density function, and the magnitude of the kernel is not very important – what matters is the relative changes in density, not the function’s absolute value. See an example of this in the spectral plot shown in Fig. 1. The format of Fig. 1 is used for all other spectral plots in this paper.

Changes in the graph’s communities can then be seen as changes in the density function. The density of a cluster is proportionate to the magnitude of the density function at the peak (i.e., the cluster centroid). Community growth and contraction can be seen by points traversing community boundaries (i.e., separatrices). Birth and death correspond to the emergence or disappearance of a peak in the density function. Merging and splitting correspond to the merging and splitting, respectively, of the density function peaks. This splitting and merging correspond very closely to pitchfork bifurcations in dynamical systems; more precisely, the pitchfork bifurcation happens to the gradient flow $\dot {\mathbf {x}} = \nabla f$. Birth and death also correspond to pitchfork bifurcations, but this is not as immediately obvious. It is a corollary of the Poincaré-Hopf theorem: creating a new maximum results in the creation of additional saddle points and/or minima (Domokos et al. 2012). To identify death, merging, or splitting, we can track the Hessian of *f*. If it becomes singular at a point, that is an indication of a potential bifurcation there. Birth may be identified in the same way, but searching the space for such a phenomenon may be more difficult than simply tracking known maxima and monitoring the Hessian at those points.

Once the spectrum has been plotted, techniques such as *k*-means clustering can identify communities. This should produce a sufficient approximation of the separatrices between maxima. However, if two eigenvectors are used, it may even be easier to identify communities visually.

## Computational experiments

### Implementation and testing procedure

To demonstrate our methods, we initially created a series of graph snapshots using a synthetic graph process. The dataset was created by generating two Erdős-Rényi (ER) random graphs with 100 nodes each, as representing distinct communities, with edge probabilities of *p*_*E*_=0.15 for both. We then began connecting the nodes belonging to the two communities through an inter-community edge probability of *p*_*int*_≪*p*_*E*_; we increased *p*_*int*_ all the way to *p*_*E*_ to simulate the distinct communities merging. Once the merger was complete, we gradually decreased *p*_*int*_ to simulate the splitting of a large community into smaller ones.

To test our methods on real-world data, we used proteomics data produced by Mitchell et al. (2013). Networks were produced by identifying subnetworks of upregulated proteins (*p*<0.05 and fold change > 1.5 compared to uninfected mocks) from the overall human protein-protein interaction network (Keshava Prasad et al. 2008). The network data indicates time-varying linkages between different proteins in human lung epithelial cells that have been infected by the Severe Acute Respiratory Syndrome corona virus (SARS-CoV). The proteomics network formed a relatively sparse, highly disconnected graph of 576 nodes, and we used the data snapshots at *t*=24,30,36,48,54,60, and 72, where *t* is the number of post-infection hours. Because this graph is disconnected (and severely so), we use the bias approach described in the “Disconnected graphs” section.

We implemented our methods in Python, making particular use of the matrix exponential and logarithm functions in the SciPy package. To evaluate the interpolation and averaging results for the synthetic network, we recorded connectivity measurements, spectral snapshots from interpolated and averaged Laplacians, and the total number of communities in the interpolated and averaged Laplacians. To measure connectivity, we used the logarithm (for scaling purposes) of the product of the non-zero Laplacian eigenvalues as mentioned in the “Graph interpolation and averaging” section. For the spectral snapshots, we used the eigenvectors corresponding to the first two non-trivial eigenvalue to produce plots as described in the “Dynamic spectral clustering” section. These snapshots provided an evaluation that was more qualitative than quantitative. We then used the Louvain method to perform community detection. The graph snapshots are provided in Additional file 1, and the code implementing the methods is provided in Additional file 2.

The spectral snapshots and connectivity measurements were not as useful for the proteomics network because the proteomics network was highly disconnected, but the Louvain method was still applicable for community detection. To investigate the interpolation and averaging of community structure for this network, we tracked the total number of communities, the total number of communities with at least five members, community similarity, and graph energy. Because the network was highly disconnected, the Louvain method produced many small or single-member communities. Tracking the number of communities above a certain size helped to reduce the amount of noise due to that effect. By community similarity, we mean not just the number of communities but the composition of those communities as well. It can be difficult to measure the degree of similarity between two graphs’ community structures when there are many communities and the community labelling is not consistent, but we can look at the pairwise similarity with the Rand index (Rand 1971).

The Rand index works by using a baseline or ground truth case, considering every distinct pair of nodes, and determining whether or not they are in the same community. It then looks at these same pairs in another graph of interest. If, for a given pair of nodes, the nodes are either in the same community as each other in both graphs or not in the same community as each other in both graphs, that pair gets a score of 1; otherwise they get a score of 0, indicating a dissimilarity between the community structures of the two graphs. Summing the results over all pairs and dividing by the number of pairs yields a score between 0 and 1, where 1 indicates that the two graph’s community structures are identical. The smaller the value, the less similar the structures are.

Given that we had no ground truth between the data snapshots, we instead looked at the changes in this metric from one snapshot to the next. Ideally, there would be a steady change in this value between points – a sawtooth pattern over the course of the whole interpolation – as we measured how the interpolation differed from the most recent data snapshot. Finally, to measure network connectivity, we used graph energy instead of a Laplacian eigenvalue product. The energy of a graph, *E*, is defined as the sum of the absolute values of the eigenvalues of the adjacency matrix. Given that it is bounded by the number of edges, *m*, in an unweighted graph (Brualdi 2006), we can also use it to bound the number of edges:

14 $$ 2 \sqrt{m} \leq E \leq 2m \Rightarrow \frac{1}{2} E \leq m \leq \frac{1}{4} E^{2}  $$

and thus it gives us information about both graph spectra and graph connectivity.

For both sets of data, we used thresholding on the edge weights to get unweighted graph equivalents. This procedure, and especially the threshold value used, was more impactful on the proteomics data than on the synthetic data.

### Synthetic graph results

**Fig. 2:**
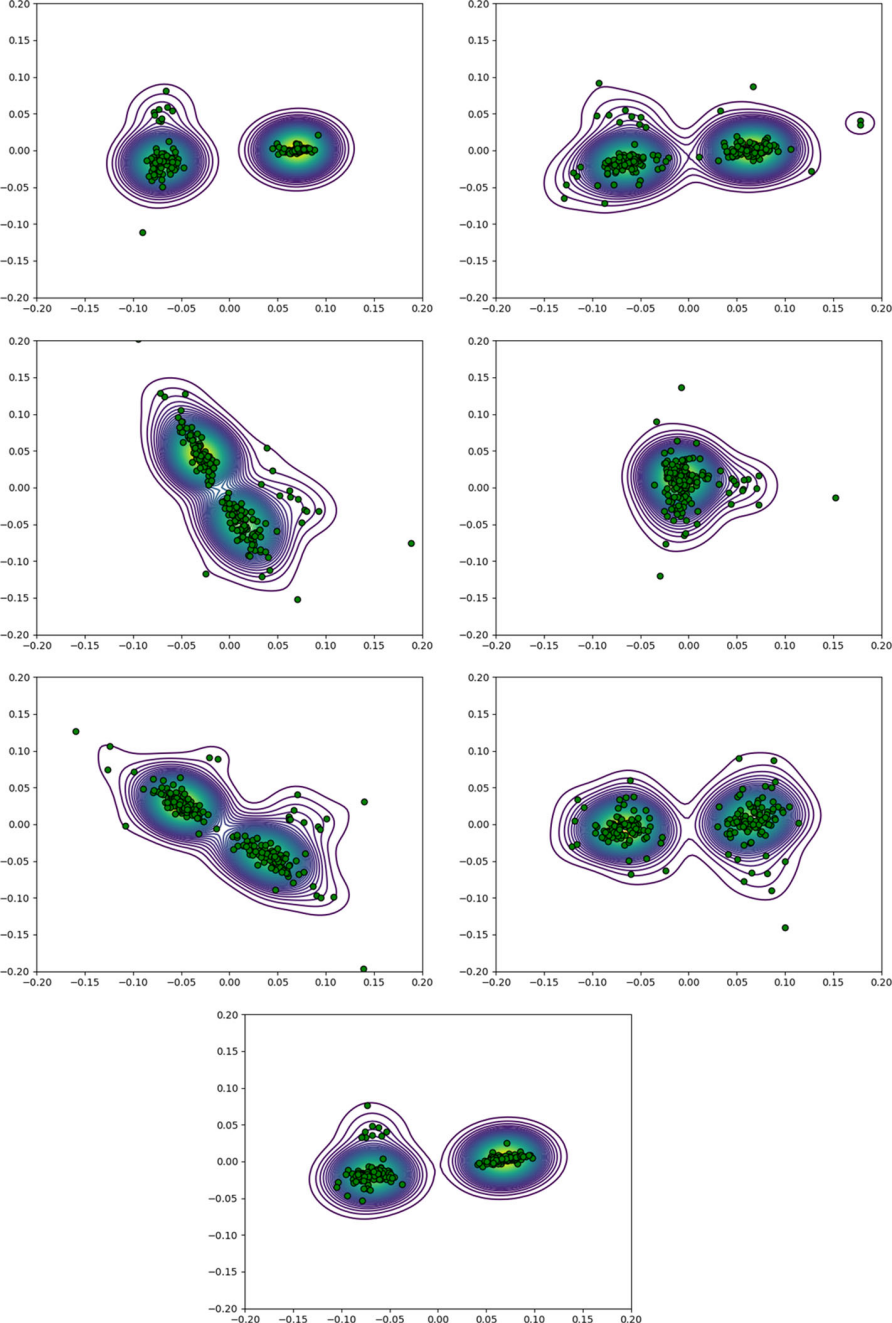
Synthetic graph spectral plots, frames 1-7. The spectral plots of the synthetic data snapshots are presented in order from left to right, and top to bottom. They show two communities that are stable and separate except for the merger shown in the fourth frame. There are also nodes that do not associate closely with any community at various points in time

**Fig. 3:**
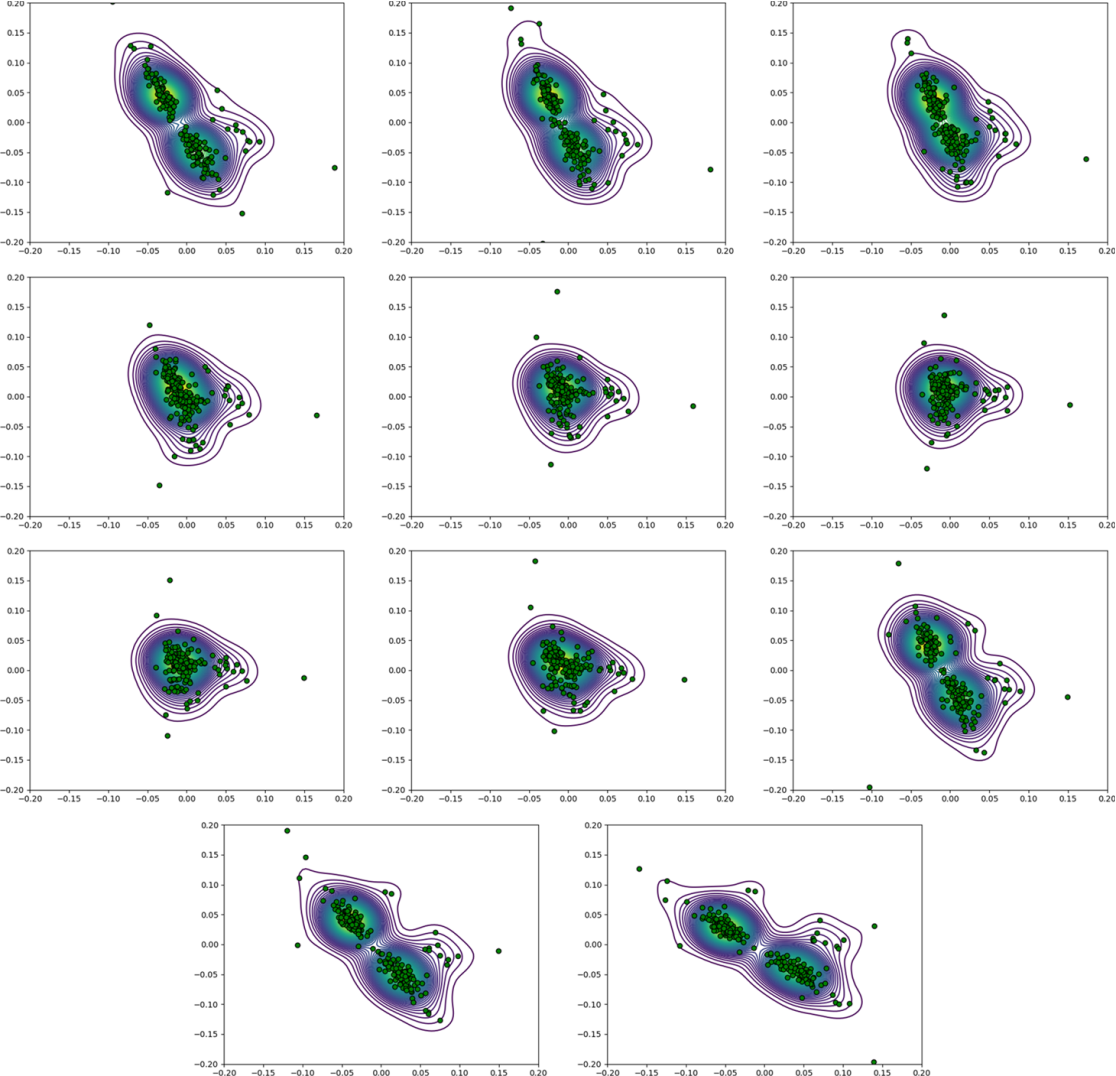
Synthetic graph interpolation. The interpolation’s frames are presented from left to right, and top to bottom. At the top left, the first frame is the third data snapshot, the sixth frame is the fourth data snapshot, and the eleventh frame is the fifth data snapshot; the interpolated frames are taken at evenly spaced time intervals between the data snapshots. The interpolated frames show a clear progression of community merging and splitting as well as some outliers that do not seem strongly attached to any community


The graph spectral snapshots are shown in Fig. 2, and we can clearly see the expected merger and separation of two communities there. We can now interpolate from the third to the fourth data snapshot and then from fourth to the fifth data snapshot to further investigate this community merger and separation. Snapshots from the AI geodesic interpolation are shown in Fig. 3; the results from the linear and LE geodesic interpolations were almost identical with these. Increasing the temporal resolution would become increasingly cumbersome for presentation in a printed format. However, the method does lend itself well to video presentations of the dynamic community behaviour (see Additional file 3 for an example).

Additional file 3: A video of the spectral plots created with the AI geodesic interpolation on the synthetic graph data progressing through the data snapshots in order from the initial to the final frame. (MP4 1966 kb)


**Fig. 4:**
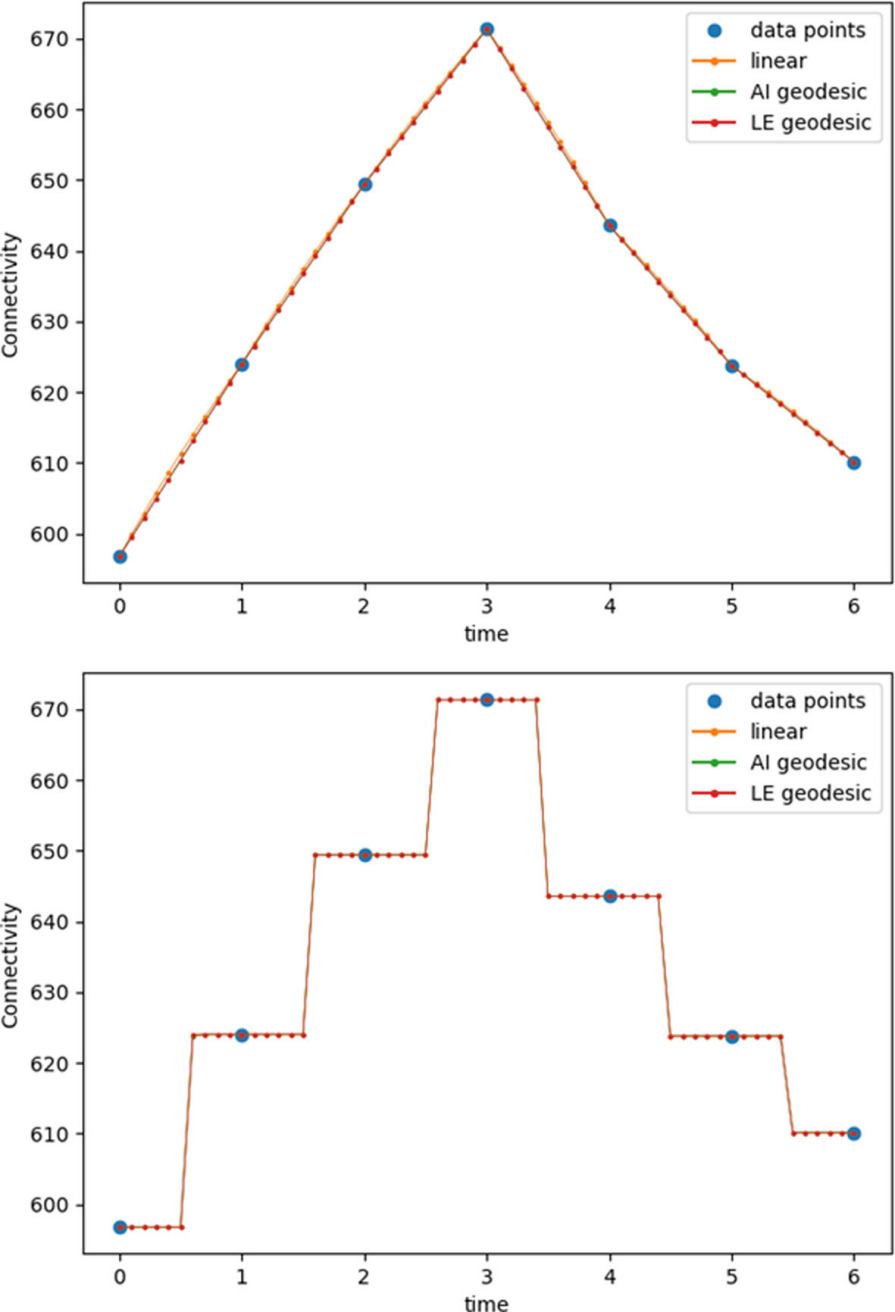
Logarithm of product of non-zero eigenvalues over time, synthetic graphs. The connectivity results are shown for the interpolations that do not use a threshold (*top*) and the interpolations that use a threshold of 0.5 (*bottom*)

**Fig. 5:**
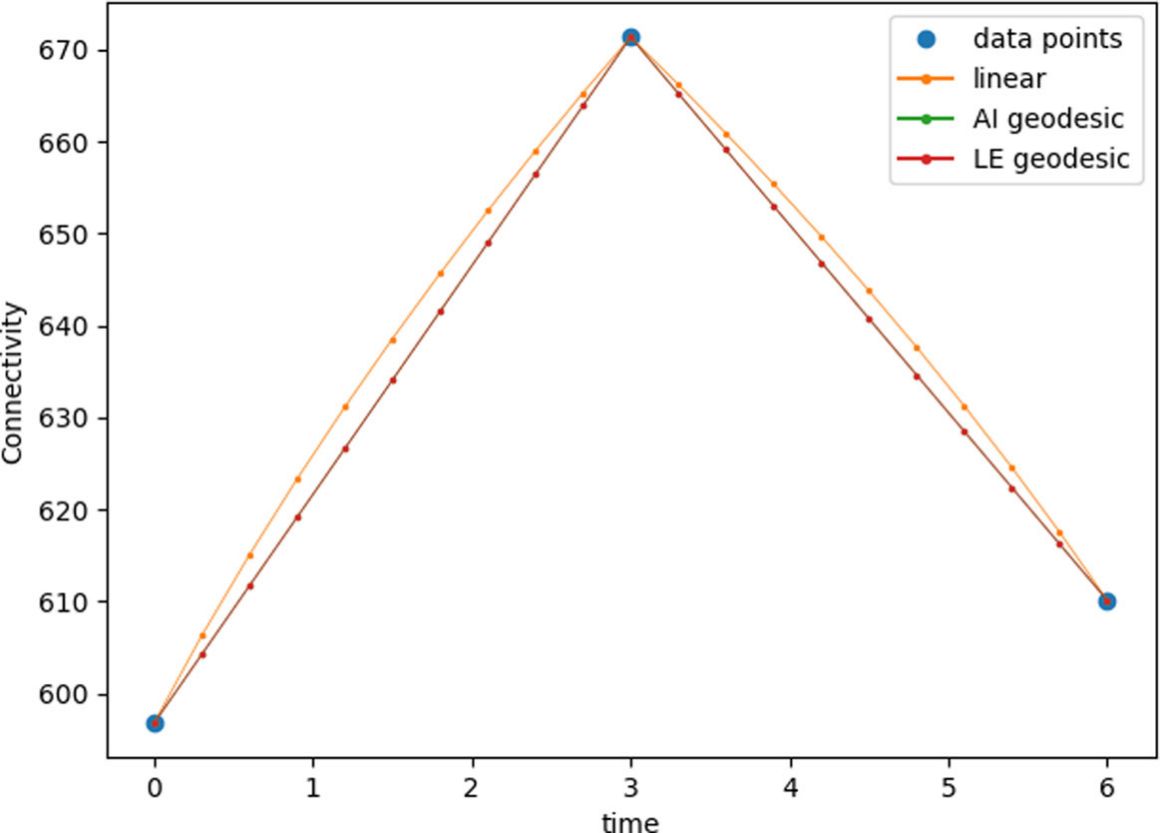
Logarithm of product of non-zero eigenvalues over time with longer interpolation window (no threshold), synthetic graphs. Interpolating between graphs that are ‘farther apart’ leads to a more apparent distinction between the geodesic and linear interpolations. The AI and LE geodesic are still indistinguishable with regards to the connectivity measure, however

In Fig. 4, we can see how the graph connectivity changes over time. The geodesic curves both interpolate the eigenvalue product linearly between points, whereas the linear interpolation is slightly concave. For this dynamic graph, the data points are relatively close to each other, and thus the geodesic and linear interpolations are very similar. If we interpolate between *t*=0, *t*=3, and *t*=6, we can see the distinction more clearly, as in Fig. 5.

Thresholding gives us a piecewise constant graph. The graph dynamics consist of an edge addition phase followed by an edge subtraction phase, so the thresholding parameter simply determines when that entry flips from 0 to 1 (or vice versa). If we were to use a finer time resolution, we might see a slight difference between the linear and geodesic interpolations with respect to when this transition happens, but the basic behaviour would remain the same.

In performing community detection, we found that the geodesic interpolations produced adjacency matrices with negative entries. Almost all of these entries were on the order of 0.001 to 0.01, and none were larger than 0.1. Negative edges need not be a barrier for community detection (e.g., see Traag and Bruggeman (2009)), but they can cause problems for the Louvain method, so in doing community detection, we simply set these entries to 0. This was only necessary for community detection on graphs that did not use thresholding. When using a threshold, any value equal to or below the threshold, including a negative value, was set to 0.

**Fig. 6:**
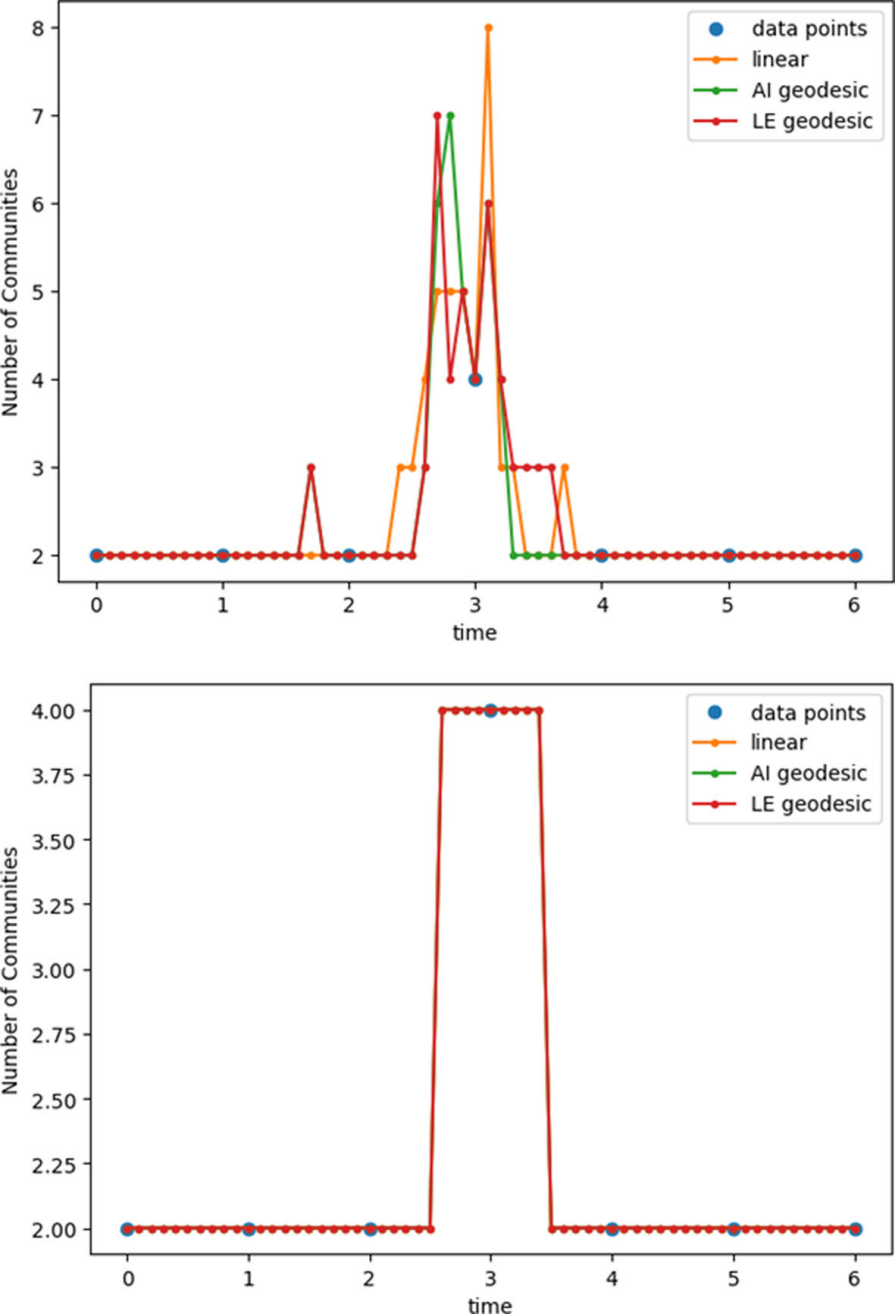
Communities in interpolated synthetic graphs. When no threshold is applied (*top*), the Louvain method produces varying numbers of communities during the merger of the two original communities, and even the data snapshot of the merged communities shows not one but four communities; we also see some differences between the two geodesic interpolations. With a threshold (*bottom*), though, the piece-wise constant nature of the interpolation shows forth

The spectral plots showed two communities merging and splitting with some outliers along the way. We found that the Louvain method split the merged community into four, and the outliers sometimes formed very small communities of their own (Fig. 6). The difference in results between the two methods suggests that in community detection, it may be worthwhile to be able to assign an ‘unaffiliated’ status to some nodes – nodes that are not really part of any community. This kind of behaviour is what gives us, for example, the brief existence of a small community (of size 3) in the LE geodesic interpolation between *t*=1 and *t*=2. When we use a threshold, these behaviours cease, as we now have a graph that is piecewise constant in time for all three interpolations.

**Fig. 7:**
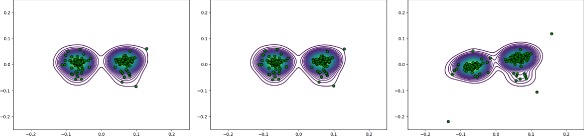
Synthetic graph average spectral plots. The spectral plots for the AI geodesic (*left*), LE geodesic (*center*), and linear (*right*) means are all very similar: the geodesic interpolations produce indistinguishable means, and the linear interpolation’s mean is only slightly different from the geodesics’

Finally, we consider the average behaviour of this graph using the mean graphs produced by each interpolation method. The spectral plots of these graphs are shown in Fig. 7, and they clearly show two distinct communities. This indicates that the merging of the two communities was only a transient effect and that the same communities re-emerged after the temporary merger. The averaging process preseved the structure that we designed the dynamic graph to have. If the second pair of communities were significantly different from the first, then the spectrum of the average graph would not display two distinct communities so clearly.

Table 1 illustrates the similarities while highlighting the small differences between the results: the geodesic interpolations consistently have slightly higher modularity and slightly lower connectivity than the linear interpolations, but thresholding the resulting graphs reduces those differences. This is not surprising given both the propensity that linear interpolations have for increasing connectivity and the similarity of the geodesic and linear interpolations in this case.

**Table 1:** Mean graph characteristics, synthetic graphs

Mean graph	Modularity	Connectivity
Linear	0.299	633.5
Linear (thresholded)	0.300	631.8
AI geodesic	0.335	631.3
AI geodesic (thresholded)	0.305	630.5
LE geodesic	0.336	631.3
LE geodesic (thresholded)	0.304	630.7

### Proteomics network results

In interpolating the proteomics network data, we again obtained negative adjacency matrix entries (around 5% of the total entries). The AI geodesics produced far fewer such entries than the LE geodesics (by an order of magnitude), and the AI entries were usually smaller. Of the negative entries, the largest was -0.16, but less than 1% of the negative entries had magnitudes greater than 0.01. As with the synthetic graphs, we simply set these negative entries to 0 when using the Louvain method.

**Fig. 8:**
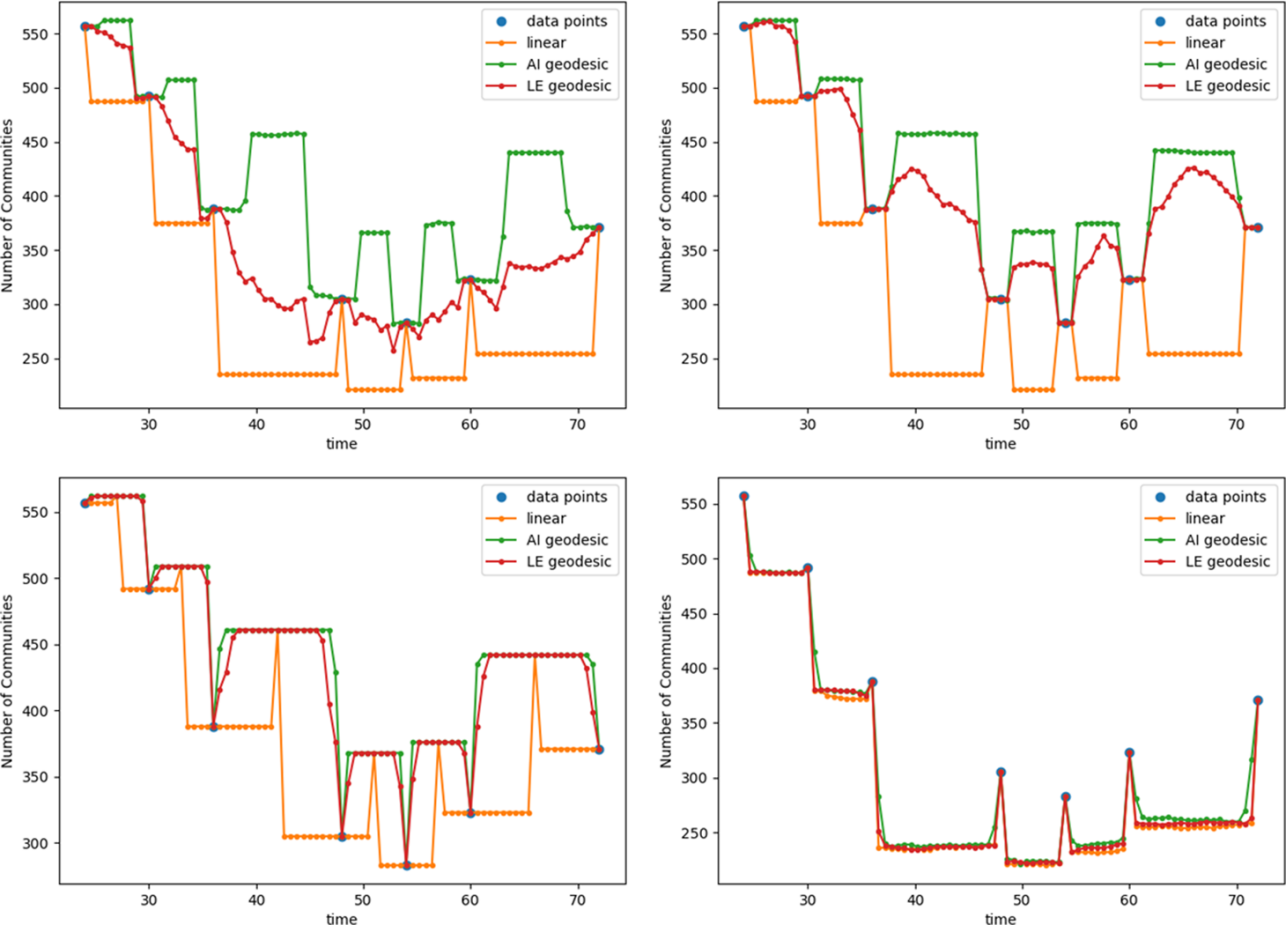
Number of communities in interpolated proteomics network. The number of communities in the interpolations using thresholds of 0.02 (*top left*), 0.1 (*top right*), and 0.5 (*bottom left*) show significant differences between the three interpolations, while using no threshold (*bottom right*) produces similar behaviour for all three

Figure 8 shows how the number of graph communities varied over time and how different thresholding levels affected those results. With no thresholding, we found that the results were too connected (i.e., not enough communities) for all three interpolation methods: after leaving a supplied data point, the number of interpolated communities would immediately drop, remain relatively constant, and shoot up upon reaching the next data point. Thresholding produced better results. Generally speaking, the AI geodesic produced too many communities while the linear interpolation produced too few, and neither produced a steady deformation from one data point to the next. The LE geodesic showed an intermediate behaviour in this regard, and a threshold of 0.02 produced best performance. The number of communities produced by the interpolation did not vary smoothly, but there was a general progression from data point to data point. Changing the threshold value had a small effect on the AI geodesic, but it did nothing to improve the linear interpolation, and using a threshold value of 0.5 actually produced an odd spike in the number of communities halfway between data points. We will return to this phenomenon later.

**Fig. 9:**
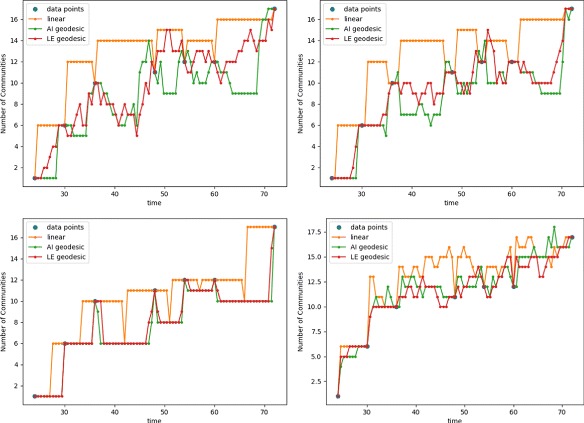
Number of communities with ≥ 5 members in interpolated proteomics network. The changes in the number of communities with at least five members were perhaps most regular when no threshold was applied (*bottom right*). Applying thresholds of 0.02 (*top left*), 0.1 (*top right*), and 0.5 (*bottom left*) produced greater differences between the linear and geodesic interpolations

In looking at Fig. 8, though, we see that there are many communities relative to the size of the graph – most of these are communities of one or two nodes that are not connected to the rest of the graph. If we only consider communities of a certain size, we can get a more accurate picture of the true community dynamics. In Fig. 9, we look only at communities that contain at least five nodes and consider how the results are affected by different threshold values. When using a threshold value, the results are somewhat similar to those in Fig. 8. In Fig. 8, there were too few communities because the graph was more connected, and we observe the effects of that increased connectivity here, too: there are fewer communities overall, but the communities that are present tend to be larger, and there are more large communities. The geodesic interpolations, on the other hand, were less connected. Therefore, they had had many small communities and relatively few larger ones; the best results came from the LE geodesic with a small threshold.

The case without thresholds was more interesting. There, the linear interpolation still often produced too many communities, but the geodesic results did not uniformly produce too few communities. The LE geodesic may have been slightly better than the AI geodesic, but they were both still producing results that looked much more reasonable than they had when we plotted the total number of communities. In fact, those results look even more regular and smooth than the thresholded results.

With some analysis, we can see why using a threshold value of 0.5 produced odd spikes in the number of communities for the linear interpolation. Let us assume that we are interpolating from adjacency matrix *A*_0_ to adjacency matrix *A*_1_. Let us denote the edges in *A*_1_ that are not in *A*_0_ with the adjacency matrix *A*_*add*_ and the edges in *A*_0_ that are not in *A*_1_ with the adjacency matrix *A*_*sub*_. Our linear interpolation from *A*_0_ at *t*=0 to *A*_1_ at *t*=1 would then be

15 $$ A(t) = A_{0} + t \left(A_{add} - A_{sub}\right)  $$

If we use a threshold *τ* such that matrix entries greater than *τ* are sent to 1 and entries less than or equal to *τ* are sent to 0, we get two possible interpolation patterns, each with three interpolated values. If *τ*<0.5, then

16 $$ A(t) = \left\{ \begin{array}{cc} A_{0} &0 \leq t \leq \tau \\ A_{0} + A_{add} & \tau < t < 1-\tau \\ A_{1} &1-\tau \leq t \leq 1 \end{array} \right.  $$

If *τ*≥0.5, then

17 $$ A(t) = \left\{ \begin{array}{cc} A_{0} &0 \leq t < 1-\tau \\ A_{0} - A_{sub} &1 - \tau \leq t \leq \tau \\ A_{1} &\tau < t \leq 1 \end{array} \right.   $$

*A*_0_−*A*_*sub*_ will be less connected than either of the interpolation end points, and if *τ*=0.5, then *A*(*t*)=*A*_0_−*A*_*sub*_ only at *t*=0.5. That is why we see that spike in the number of communities.

**Fig. 10:**
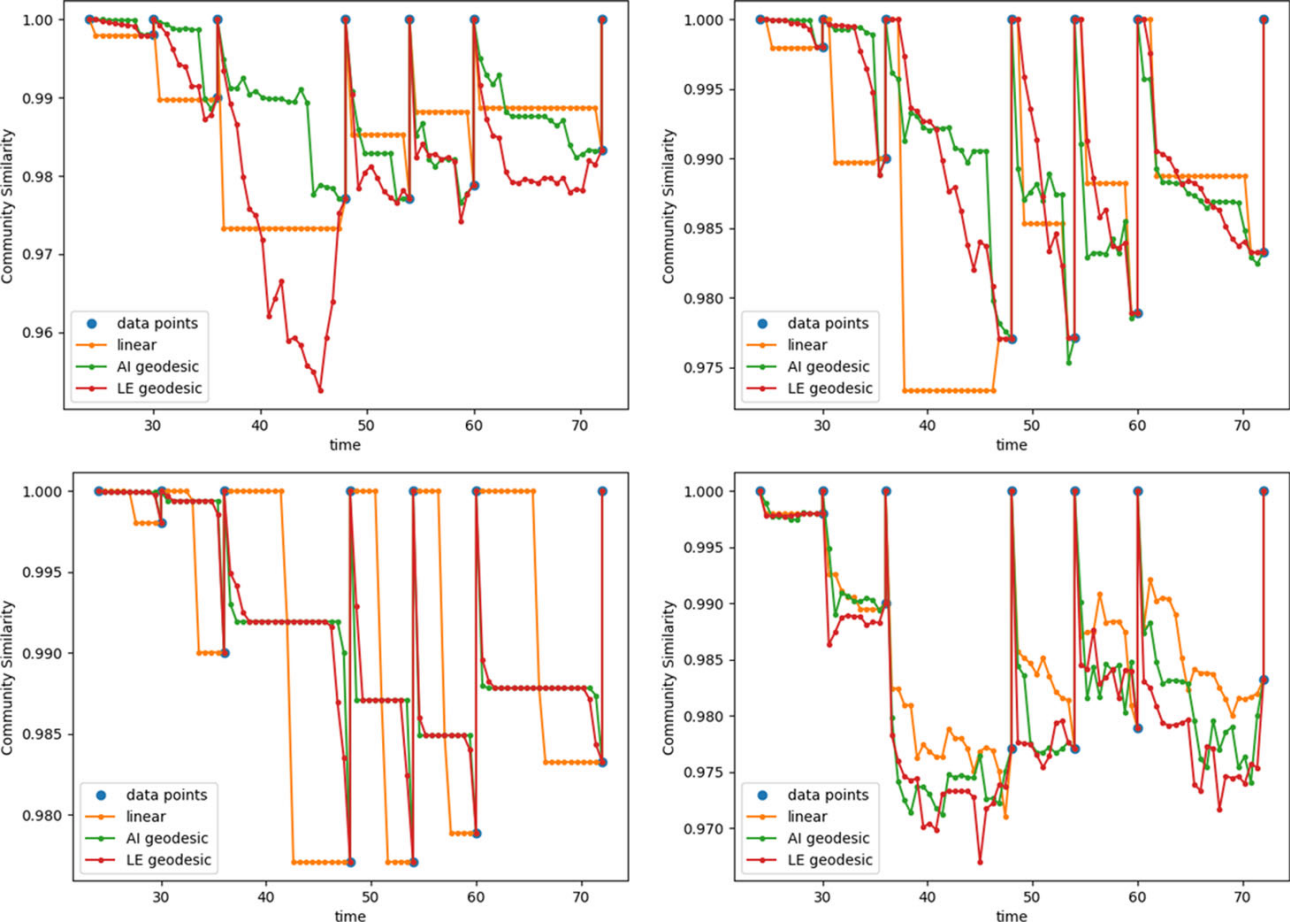
Community similarity in interpolated proteomics network. When no threshold is applied (*bottom right*), the LE geodesic displays more extreme behaviour than the AI geodesic. A threshold of 0.1 (*top right*) gives the best performance for the geodesics, a threshold of 0.02 (*top left*) produces excessive variation in the LE geodesic, and a threshold of 0.5 (*bottom left*) produces almost piecewise constant behaviour in the geodesics. The linear interpolation produces reasonable results when no threshold is applied

The community similarity results are shown in Fig. 10. With no thresholding, the linear interpolation performs best. Both of the geodesics tend to become even less similar to the previous snapshot than the snapshot they are progressing towards, resulting in a U-shape, whereas the linear interpolation has a more consistent decrease. All three interpolations, though, show a sharp decrease in similarity immediately after leaving a snapshot. Surprisingly, the LE geodesic also produces more extreme results than the AI geodesic. Thresholding produces the best result, and it does so with the LE geodesic and a threshold of 0.1. The linear interpolation once again shows its piecewise constant behaviour, but a threshold of 0.02 is no longer optimal for the LE geodesic, and the AI geodesic performs reasonably well at that threshold value.

**Fig. 11:**
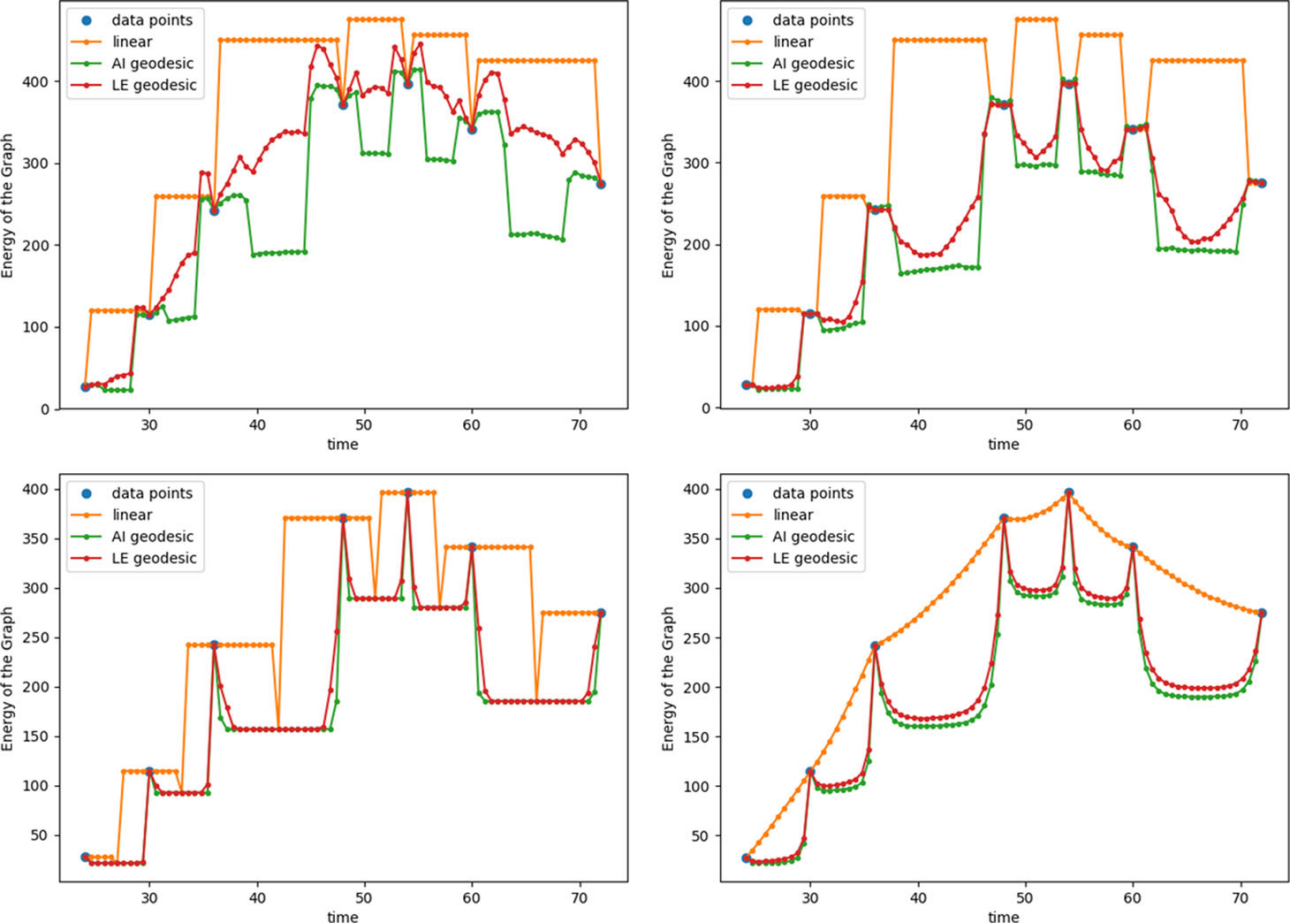
Graph energy in interpolated proteomics network. Applying thresholds of 0.5 (*bottom left*), 0.1 (*top right*), and 0.02 (*top left*) produced the same kind of trends in the interpolations’ graph energy as was the case in considering the number of communities: low-energy (i.e., less connected) graphs with the AI geodesic, high energy (i.e., more connected) graphs with the linear interpolation, and graphs of varying energy with the LE geodesic. Interpolation without a threshold (*bottom right*) gave similar performance for the geodesics

Plots of the energy of the interpolated graphs are shown in Fig. 11. When no threshold is applied, the linear interpolation produces an almost linear progression, whereas both geodesic methods go through significant minima between data points. The geodesics are designed to interpolate Laplacian eigenvalue products linearly, whereas the linear interpolation produces a linear variation in the eigenvalue sum. Linearly changing the sum produces a concave change in the determinant, as we saw in Fig. 5, and we can now see that linearly changing the product produces a convex change in the sum. The interpolations in question are being performed on the Laplacian, not the adjacency matrix, but we can see a clear connection.

When we look at the thresholded results, we see that the linear interpolation consistently produces graphs with high energy values, the AI geodesic produces graphs with low energy values, and the LE geodesic is somewhere in the middle. For the LE geodesic, the best threshold value is around 0.02, where the interpolation produces a relatively steady change in graph energy from data point to data point (unlike the linear and AI geodesic interpolations, which basically plateau between points). This is consistent with what we saw in Fig. 8 and what we know about sparsity and the different interpolations.

Finally, we can look at the average graphs calculated using the three different methods. Table 2 shows the number of communities for each of the averaged graphs, and Table 3 shows the number of communities with at least five nodes in those graphs. The average graph without thresholding showed a much higher level of connectivity than any of the data snapshots, and this was the case for all of the averaging methods. This would make sense if the community structure changed significantly from snapshot to snapshot. Thresholding the average graph produced more reasonable results, though the AI average was highly disconnected, and the linear average showed a very large change in behaviour when the threshold dropped below 0.5.

**Table 2:** Number of communities in average graph

	Threshold			
Interpolation type	0.02	0.1	0.5	None
Linear	77	77	439	78
AI geodesic	538	539	569	82
LE geodesic	495	530	565	75

**Table 3:** Number of communities with ≥ 5 members in average graph

	Threshold			
Interpolation type	0.02	0.1	0.5	None
Linear	19	19	7	19
AI geodesic	3	3	0	16
LE geodesic	7	4	1	14

Table 3 records results congruent with those in Table 2. With the linear mean graph, we see more communities with at least five members than any of the individual graph snapshots have – again, the linear interpolation produces results with increased connectivity. The Riemannian mean graphs without thresholds produce more reasonable numbers of communities, but applying a threshold to the geodesic means severely reduces those numbers. The most reasonable result with a threshold seems to be the LE mean with a threshold of 0.02 or the linear mean with a threshold of 0.5.

Next, we can look at the average difference in community assignment between the mean graphs and the data snapshots in Table 4. The linear mean performs better than the others when no threshold is used, but with a threshold, the best results come from the Riemannian means (which are almost identical). These values are quite high – both here and in the interpolation results shown in Fig. 10 – and this is likely due to the large number of unconnected nodes.

**Table 4:** Average similarity in community assignment

	Threshold			
Interpolation type	0.02	0.1	0.5	None
Linear	0.948	0.948	0.989	0.945
AI geodesic	0.990	0.990	0.990	0.932
LE geodesic	0.989	0.990	0.990	0.905

The basic trends in the numbers of communities are reflected in the graph energies recorded in Table 5: the linear averages have very high energy and the geodesic averages have very low energies, with the LE averages’ energies slightly higher than the AI averages’. What is somewhat surprising, though, is the difference in graph energies between the non-thresholded means – the numbers of communities in each are similar, but the linear average has an energy roughly an order of magnitude higher than the Riemannian averages. The energy of the linear mean without thresholding or with a threshold of 0.5 seem to be the most reasonable values.

**Table 5:** Graph energy in average graph

	Threshold			
Interpolation type	0.02	0.1	0.5	None
Linear	647.2	647.2	194.2	229.6
AI geodesic	55.9	49.1	11.5	19.2
LE geodesic	93.7	55.9	14.9	25.2

In concluding our observations about these averages, we note that the weights on the linear average graph will all have weights that are multiples of 1/7 (because there are seven data points provided), and thus there will be no difference in results for any two thresholds that lie between $\frac {n}{7}$ and $\frac {n+1}{7}$. This explains why the results for threshold values of 0.02 and 0.1 are the same for the linear average, for example. The geodesic interpolations provide no such structure, and our results here would suggest that low thresholds are generally required to get good results out of the geodesic interpolations.

## Discussion and future work

### Interpolation error

In the Appendix, we have provided error bounds for each geodesic interpolation in terms of distance on their respective manifolds. The actual error incurred will depend on the problem in question, though. That kind of error, or even entry-wise error, may not be the most important kind of error to consider for our purposes here, however. Rather, we may care most about the community structure.

Based on our community-related metrics (connectivity and similarity), the LE geodesic, with a threshold for the proteomics data, performed the best. The AI geodesic was too sparse and disconnected, while the linear interpolation was too connected (as expected). The optimal choice of threshold value depended on the metric being considered: 0.1 was by far the best when considering community similarity, but 0.02 was better for the other metrics under consideration. In general, the optimal threshold value will likely depend on the problem in question and the quantities of interest, but we found that the LE geodesic responded to changes in the threshold value more readily than the AI geodesic did.

**Fig. 12:**
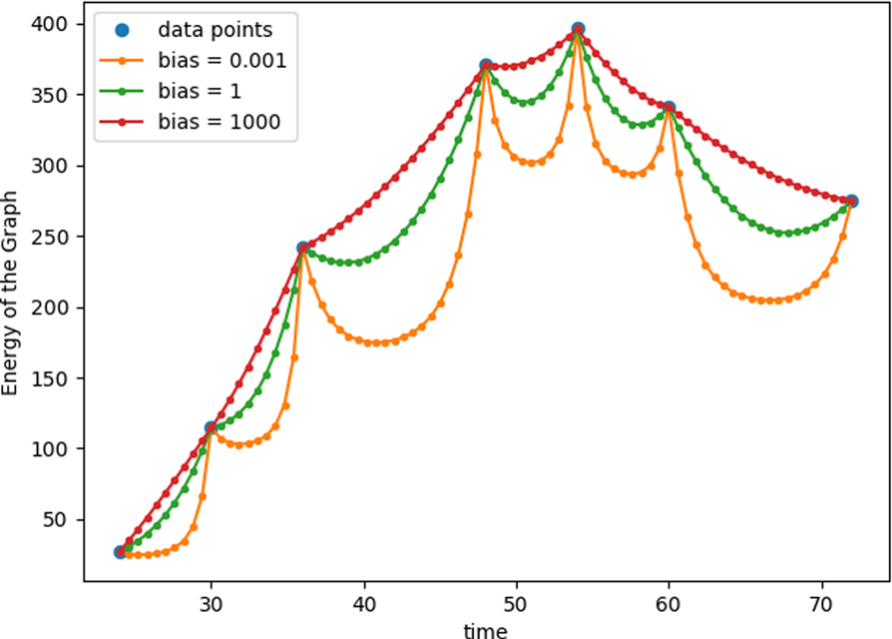
Graph energy in interpolated proteomics network, LE geodesic with varying bias values. Using a bias value of 1.0 essentially produces an average of the geodesic and linear interpolations; a bias value of 1000 produces results similar to the linear interpolation, and a bias value of 10^−3^ produces results similar to those obtained with a bias value of 10^−6^

In this paper, we used the same bias value for all of the proteomics interpolations (10^−6^), but as mentioned in the “Disconnected graphs” section, increasing the bias value caused the geodesic interpolation to approach the linear one. Figure 12 shows an example of this where increasing the bias term causes the LE geodesic to behave more and more like the linear interpolation (compare with Fig. 11). Future work may involve experimenting with different bias terms to find a happy medium between the linear and pure geodesic interpolations.

One concern about the geodesic interpolations is the transient edges that they produce– edges that do not exist in either end point but emerge and disappear during the interpolation process. The weights on these edges were small, but they could be positive or negative, and they arose in both the synthetic and proteomics data, so they are not simply an artefact of using the bias addition approach to deal with disconnected graphs. Moreover, using a low threshold means that some of these edges may not disappear when that threshold is applied, and therefore they may affect the community structure of the graph. Using a larger bias value to more closely approximate a linear interpolation may ameliorate the problem, but it would be valuable to look in more detail at why these transients occur and how to interpret them from a graph theoretic perspective. For example, does it make sense to say that the ‘shortest’ or ‘least energetic’ path from one graph snapshot to another might involve some transient edges? From the perspective of the manifold geometry, it clearly does, as the shortest path between two points *is* a geodesic, but it is not clear if the same holds true purely from a network perspective.

In short, the geodesic interpolations are not perfect, and there are still unanswered questions, but it is nonetheless clear that linear interpolation is not well suited to graph interpolation if the ultimate goal is community detection. When using a threshold, linear interpolation will always produce a piecewise constant result consisting of three phases. Without thresholding, the linear interpolation inflates overall graph connectivity, and the greater the difference between the two graphs, the greater the inflation. As an extreme example, consider interpolating between a graph with adjacency matrix *A* to a graph with adjacency matrix 1−*A*. The result ‘halfway’ between them would be a fully connected graph with edge weights of 0.5. These issues are particularly prominent when calculating averages over multiple graphs.

Perhaps most saliently for our purposes here, the linear interpolation did not produce steady changes in the community structure between data points – the proteomics data showed that the linear interpolation almost always had markedly fewer communities than the data points it connected. The AI geodesic produced transient edges that were smaller in magnitude and fewer in number than the LE geodesic, but it was also more expensive and produced graphs that were too sparse (e.g., too few communities); the LE geodesic used a similar approach but produced better results when combined with a threshold. Similar trends held true, generally speaking, for the mean graphs as well.

### Computational cost and supporting methods

Currently, the computational cost of geodesic interpolation is high because it requires calculating matrix functions like the exponential and logarithm. The LE geodesic is noticeably faster than the AI geodesic in calculating interpolated points, though, due to the fractional matrix powers used in the former but not the latter. Furthermore, the average graph is significantly easier to calculate for the LE geodesic because it has a closed-form expression, whereas the AI geodesic requires an iterated numerical solution. These computational costs are not prohibitive for graphs with hundreds of nodes, but for much larger graphs – say, on the order of 10^6^ nodes – the computational cost could render our methods infeasible. One possible approach would be to project the graph Laplacians to a lower-dimensional space, perform the interpolation there, and then project back to the original space with some kind of low-rank or sparsity criterion; Riemannian optimization on matrix manifolds could be useful for determining an optimal low-rank projection (Vandereycken 2013).

Another option would be to use graph spectral sparsification (Batson et al. 2013) to produce sparse graphs that approximate the spectrum of the original graph. We would then perform the interpolation on those sparse graphs. Given the close relationship between geodesics and spectral properties, this approach may be better-suited to the geodesic interpolations than to the linear interpolation. Either way, it should be possible to come up with an error bound, in terms of the distance between the approximate and true solutions, that relates to the approximation used.

As an alternative to thresholding, it may also be possible to identify the Laplacians of unweighted graphs that are ‘closest’ to the geodesic trajectory and use them to define a kind of discrete trajectory of unweighted graphs that most closely approximates the geodesic between two unweighted graphs. This could potentially be more accurate than simply thresholding the adjacency matrix entries.

### Additional interpolation and clustering methods

Our present interpolation methods match the supplied data points exactly, but the transitions from one interpolation to another are not smooth. It may be valuable to develop more sophisticated interpolation methods that will enforce smoothness, such as polynomial and spline interpolation, using the form of the geodesic interpolations. We may not want to match the supplied graph snapshots exactly, though. Instead, we may need to come up with an approximating curve for noisy data. It is possible to define a geodesic that minimizes the sum of squared distances between it and a set of time-indexed data (much like a linear least-squares regression). We could then solve for the regression coefficients in a manner similar to the calculation of the geodesic mean. Both higher-order interpolations and least-squares interpolations are possible for the AI and LE geodesics, but they may be easier to derive and computationally cheaper for the LE versions than the AI versions. Regardless of which is used, though, the geometries in which the interpolations are embedded would ensure that the Laplacians remain positive-semidefinite and thus representative of real graphs.

There is also the option of using other Laplacians (e.g., a normalized Laplacian). Some of these Laplacians have spectral properties, such as bounded eigenvalues, that may induce better interpolation behaviour. If these Laplacians also have non-constant nullspaces, though, that would add complexity to the interpolation procedure. This would not be a significant hurdle for piecewise geodesic interpolation, but it may be problematic for graph averaging and some of the interpolation expansions described in the paragraph above. We have not yet looked at this problem in detail, however.

Finally, as mentioned previously, the Riemannian framework does not require any one particular community detection method, though it may have some natural connections to spectral clustering. Future work with the framework could include comparing different static clustering methods (either analytically or computationally) to see if there are any that would be particularly well- or ill-suited to this kind of interpolation and averaging.

## Conclusions

We described and implemented Riemannian methods for interpolating between and averaging dynamic graph snapshots. Following that, we demonstrated the use of these methods on a synthetically generated dynamic graph and an experimentally produced proteomics network and compared them with entry-wise linear interpolation. The linear interpolation increased graph connectivity between interpolation points, and we showed that when a threshold is used to produce unweighted graphs from the interpolation, the entry-wise linear approach will always produce a three-phase piecewise constant result.

The geodesic interpolations created using the Riemannian methods produced graphs with linearly varying connectivity when applied to connected graph snapshots and produced decreased connectivity between interpolation points when applied to disconnected graph snapshots. We found that using a low threshold on the edge weights improved our results on the disconnected graphs. However, these interpolations produced transient edges (with small positive and negative weights). One area of future work will be to investigate why this behaviour occurs and interpret it in graph theoretic terms. Choosing larger bias values when applying these methods to disconnected graphs may improve the quality of the interpolation, from the perspective of graph connectivity, and it may also reduce the presence of transient edges as well.

Other significant next steps for this work include developing techniques for applying our work to significantly larger graphs and expanding upon our current interpolation methods to produce the Riemannian analogues of polynomial interpolation, spline interpolation, and least-squares regression.

## Appendix

### Error estimate calculations

For 1-D linear interpolation, there is a well-defined error bound for the interpolation: a linear interpolation of *f*(*x*) from *x*_0_ to *x*_1_ has an error bound of

18 $$ \frac{1}{8} \left(x_{1}-x_{0}\right)^{2} \max \left| f^{\prime} (\xi) \right|  $$

We can then consider the Euclidean distance between a trajectory **x**(*t*) and its approximation **y**(*t*), *t*∈[0,1], from which we can calculate an error **z**(*t*):

19 $$\begin{array}{*{20}l} \mathbf{z} (t) &= \mathbf{x}(t) - \mathbf{y}(t), \ \mathbf{z} (0) = \mathbf{z}(1) = \mathbf{0} \end{array} $$

20 $$\begin{array}{*{20}l} \left\| \mathbf{z} \right\|^{2} &\leq \frac{1}{8} \max \limits_{\tau \in \left[0,1\right]} \frac{d^{2}}{dt^{2}} \left(\left\| \mathbf{z} \right\|^{2}\right) \end{array} $$

21 $$\begin{array}{*{20}l} \frac{d^{2}}{dt^{2}} \left(\left\| \mathbf{z} \right\|^{2}\right) &= 2 \ddot{\mathbf{x}} \cdot \mathbf{z} - 2 \ddot{\mathbf{y}} \cdot \mathbf{z} + 2 \left\| \dot{\mathbf{z}} \right\|^{2} \end{array} $$

22 $$\begin{array}{*{20}l} \left\| \mathbf{z} \right\|^{2} &\leq \frac{1}{8} \max \limits_{\tau \in \left[0,1\right]} \left(2 \ddot{\mathbf{x}} \cdot \mathbf{z} - 2 \ddot{\mathbf{y}} \cdot \mathbf{z} + 2 \left\| \dot{\mathbf{x}} - \dot{\mathbf{y}} \right\|^{2} \right)  \\ &\leq \frac{1}{4} \max \limits_{\tau \in \left[0,1\right]} \left(\left|\ddot{\mathbf{x}} \cdot \mathbf{z}\right| + \left|\ddot{\mathbf{y}} \cdot \mathbf{z}\right| + \left\| \dot{\mathbf{x}} - \dot{\mathbf{y}} \right\|^{2} \right) \end{array} $$

We cannot say that a linear interpolation will always have the smallest amount of error, but a linear interpolation would have $\ddot {\mathbf {y}} = 0$, so we would expect it to have a smaller error bound than an arbitrary nonlinear interpolation (i.e., one not using higher-order derivative information).

#### AI Geodesic

We end up with a similar result in considering the distance between a dynamic graph trajectory *X*(*t*) through the positive-definite subspace of the Laplacian and an AI geodesic interpolation *Y*(*t*) between *X*(0)=*R*_0_ and *X*(1)=*R*_1_:

23 $$\begin{array}{*{20}l} d^{2} \left(X,Y\right) &= \left\| \ln X^{-\frac{1}{2}} Y X^{-\frac{1}{2}} \right\|^{2} = \text{tr} \left(\Omega^{2}\right) \end{array} $$

24 $$\begin{array}{*{20}l} \Omega &= \ln \Psi \Leftrightarrow \exp \Omega = \Psi = X^{-\frac{1}{2}} Y X^{-\frac{1}{2}} \end{array} $$

25 $$\begin{array}{*{20}l} \frac{d}{dt} \left(d^{2} \left(X,Y\right)\right) &= \frac{d}{dt} \left(\text{tr} \left(\Omega^{2}\right) \right) = 2 \text{tr}\left(\Omega \dot{\Omega}\right) \end{array} $$

26 $$\begin{array}{*{20}l} \frac{d^{2}}{dt^{2}} \left(d^{2} \left(X,Y\right)\right) &= 2\frac{d}{dt} \left(\text{tr}\left(\Omega \dot{\Omega}\right)\right) \end{array} $$

*Ω* and *Ψ* commute with each other and with powers of each other (including negative powers) because they have the same eigenvectors. Traces of matrix products are also constant under cyclic permutations of those products. We will use these properties to derive an expression for $\text {tr}\left (\Omega \dot {\Omega }\right)$ using this matrix commutivity and Greene’s results on traces of matrix products (Greene 2014):

27 $$\begin{array}{*{20}l} \Omega \frac{d}{dt} \left(\exp \Omega \right) \exp \left(- \Omega\right) &= \Omega \dot{\Psi} \Psi^{-1} \end{array} $$

28 $$\begin{array}{*{20}l} \text{tr} \left(\Omega \frac{d}{dt} \left(\exp \Omega \right) \exp \left(- \Omega\right) \right) &= \text{tr} \left(\Omega \sum \limits_{k=0} \frac{1}{k!} \sum \limits_{r=0}^{k-1} \Omega^{r} \dot{\Omega} \Omega^{k-r-1} \exp \left(-\Omega\right) \right)  \\ &= \sum \limits_{k=0} \frac{1}{k!} \sum \limits_{r=0}^{k-1} \text{tr} \left(\Omega \Omega^{r} \dot{\Omega} \Omega^{k-r-1} \exp \left(-\Omega\right) \right)  \\ &= \sum \limits_{k=0} \frac{1}{k!} \sum \limits_{r=0}^{k-1} \text{tr} \left(\Omega \dot{\Omega} \Omega^{k-r-1} \exp \left(-\Omega\right) \Omega^{r} \right)  \\ &= \sum \limits_{k=0} \frac{1}{k!} \sum \limits_{r=0}^{k-1} \text{tr} \left(\Omega \dot{\Omega} \Omega^{k-r-1} \Omega^{r} \exp \left(-\Omega\right) \right)  \\ &= \sum \limits_{k=0} \frac{1}{k!} \sum \limits_{r=0}^{k-1} \text{tr} \left(\Omega \dot{\Omega} \Omega^{k-1} \exp \left(-\Omega\right) \right)  \\ &= \text{tr} \left(\Omega \dot{\Omega} \left(\sum \limits_{k=1} \frac{1}{k!} k \Omega^{k-1}\right) \exp \left(-\Omega\right) \right)  \\ &= \text{tr} \left(\Omega \dot{\Omega} \left(\sum \limits_{k=1} \frac{1}{\left(k-1\right)!} \Omega^{k-1}\right) \exp \left(-\Omega\right) \right)  \\ &= \text{tr} \left(\Omega \dot{\Omega} \exp \Omega \exp \left(-\Omega\right) \right) = \text{tr}\left(\Omega \dot{\Omega} \right) \end{array} $$

29 $$\begin{array}{*{20}l} \Rightarrow \text{tr}\left(\Omega \dot{\Omega}\right) &= \text{tr} \left(\Omega \dot{\Psi} \Psi^{-1}\right) \end{array} $$

30 $$\begin{array}{*{20}l} \Omega \dot{\Psi} \Psi^{-1}&= \Omega \left(\frac{d}{dt} \left(X^{-\frac{1}{2}}\right) Y X^{-\frac{1}{2}} + X^{-\frac{1}{2}} \dot{Y} X^{-\frac{1}{2}} + X^{-\frac{1}{2}} Y \frac{d}{dt} \left(X^{-\frac{1}{2}}\right) \right) X^{\frac{1}{2}} Y^{-1} X^{\frac{1}{2}}  \\ &= \Omega \frac{d}{dt} \left(X^{-\frac{1}{2}}\right) X^{\frac{1}{2}} + \Omega X^{-\frac{1}{2}} \dot{Y} Y^{-1} X^{\frac{1}{2}} + \Omega X^{-\frac{1}{2}} Y \frac{d}{dt} \left(X^{-\frac{1}{2}}\right) \Psi^{-1} \end{array} $$

31 $$\begin{array}{*{20}l} \text{tr} \left(\Omega \dot{\Psi} \Psi^{-1}\right) &= \text{tr} \left(\Omega \frac{d}{dt} \left(X^{-\frac{1}{2}}\right) X^{\frac{1}{2}}\right) + \text{tr} \left(\Omega X^{-\frac{1}{2}} \dot{Y} Y^{-1} X^{\frac{1}{2}} \right)  \\ & \quad + \text{tr} \left(\Omega X^{-\frac{1}{2}} Y \frac{d}{dt} \left(X^{-\frac{1}{2}}\right) \Psi^{-1}\right) \end{array} $$

Since *Ψ*^−1^ and *Ω* commute,

32 $$\begin{array}{*{20}l} \text{tr} \left(\Omega X^{-\frac{1}{2}} Y \frac{d}{dt} \left(X^{-\frac{1}{2}}\right) \Psi^{-1}\right) &= \text{tr} \left(\Psi^{-1} \Omega X^{-\frac{1}{2}} Y \frac{d}{dt} \left(X^{-\frac{1}{2}}\right)\right)\\ &= \text{tr} \left(\Omega \Psi^{-1} X^{-\frac{1}{2}} Y \frac{d}{dt} \left(X^{-\frac{1}{2}}\right)\right)\\ &= \text{tr} \left(\Omega X^{\frac{1}{2}} Y^{-1} X^{\frac{1}{2}} X^{-\frac{1}{2}} Y \frac{d}{dt} \left(X^{-\frac{1}{2}}\right)\right) \\ &= \text{tr} \left(\Omega X^{\frac{1}{2}}\frac{d}{dt} \left(X^{-\frac{1}{2}}\right)\right) \end{array} $$

Since $X^{-\frac {1}{2}} X^{-\frac {1}{2}} = X^{-1}$,

33 $$\begin{array}{*{20}l} \frac{d}{dt} \left(X^{-\frac{1}{2}}\right) X^{-\frac{1}{2}} + X^{-\frac{1}{2}} \frac{d}{dt} \left(X^{-\frac{1}{2}}\right) = \frac{d}{dt} \left(X^{-1}\right) = - X^{-1} \dot{X} X^{-1} \end{array} $$

34 $$\begin{array}{*{20}l} \Rightarrow X^{\frac{1}{2}} \frac{d}{dt} \left(X^{-\frac{1}{2}}\right) + \frac{d}{dt} \left(X^{-\frac{1}{2}}\right) X^{\frac{1}{2}} = - X^{-\frac{1}{2}} \dot{X} X^{-\frac{1}{2}} \end{array} $$

Therefore,

35 $$\begin{array}{*{20}l} \text{tr} \left(\Omega \dot{\Psi} \Psi^{-1}\right) &= \text{tr} \left(\Omega \frac{d}{dt} \left(X^{-\frac{1}{2}}\right) X^{\frac{1}{2}}\right) + \text{tr} \left(\Omega X^{-\frac{1}{2}} \dot{Y} Y^{-1} X^{\frac{1}{2}} \right) + \text{tr} \left(\Omega X^{\frac{1}{2}}\frac{d}{dt} \left(X^{-\frac{1}{2}}\right)\right) \\ &= \text{tr} \left(\Omega X^{-\frac{1}{2}} \dot{Y} Y^{-1} X^{\frac{1}{2}} \right) - \text{tr} \left(\Omega X^{-\frac{1}{2}} \dot{X} X^{-\frac{1}{2}}\right) \\ &= \text{tr} \left(\Omega X^{-\frac{1}{2}} \dot{Y} Y^{-1} X X^{-\frac{1}{2}} \right) - \text{tr} \left(\Omega X^{-\frac{1}{2}} \dot{X} X^{-\frac{1}{2}}\right) \\ &= \text{tr} \left(X^{-\frac{1}{2}} \Omega X^{-\frac{1}{2}} \dot{Y} Y^{-1} X \right) - \text{tr} \left(X^{-\frac{1}{2}}\Omega X^{-\frac{1}{2}} \dot{X}\right) \end{array} $$

For the AI geodesic interpolation (Pennec et al. 2006),

36 $$\begin{array}{*{20}l} \dot{Y}_{g} (t) &= R_{0}^{\frac{1}{2}} C^{\frac{1}{2}} \exp (Ct) C^{\frac{1}{2}} R_{0}^{\frac{1}{2}} \end{array} $$

37 $$\begin{array}{*{20}l} \dot{Y}_{g} Y^{-1}_{g} &= R_{0}^{\frac{1}{2}} C R_{0}^{-\frac{1}{2}} \end{array} $$

38 $$\begin{array}{*{20}l} C &= \ln R_{0}^{-\frac{1}{2}} R_{1} R_{0}^{-\frac{1}{2}} \end{array} $$

since *C* (and its powers) commute with exp(*Ct*). Therefore, $\dot {Y}_{g} Y^{-1}_{g}$ is constant in time. For the entry-wise linear interpolation, however, there is no closed-form expression for $\dot {Y}_{l}$ or $Y^{-1}_{l}$; *Y*_*l*_ is the positive-definite component of the interpolated Laplacian. In general, though, $\dot {Y}_{l} Y^{-1}_{l}$ will *not* be constant in time. We can then consider the second derivative of the original distance function:

39 $$\begin{array}{*{20}l}{} \frac{d}{dt} \left(\text{tr} \left(\Omega \dot{\Psi} \Psi^{-1}\right) \right) &= \text{tr}\left(\frac{d}{dt} \left(X^{-\frac{1}{2}} \Omega X^{-\frac{1}{2}}\right) \dot{Y} Y^{-1} X\right) + \text{tr} \left(X^{-\frac{1}{2}} \Omega X^{-\frac{1}{2}} \frac{d}{dt} \left(\dot{Y} Y^{-1}\right) X \right) \\ & \quad + \text{tr} \left(X^{-\frac{1}{2}} \Omega X^{-\frac{1}{2}} \dot{Y} Y^{-1} \dot{X} \right) - \text{tr}\left(\frac{d}{dt} \left(X^{-\frac{1}{2}} \Omega X^{-\frac{1}{2}} \right) \dot{X}\right)\\ &\quad - \text{tr}\left(X^{-\frac{1}{2}} \Omega X^{-\frac{1}{2}} \ddot{X}\right) \end{array} $$

For the geodesic, $\frac {d}{dt} \left (\dot {Y}_{g} Y^{-1}_{g}\right) = 0$, but this will not be the case for the entry-wise linear interpolation. Also note the recurrent $X^{-\frac {1}{2}} \Omega X^{-\frac {1}{2}}$ term: $X^{\frac {1}{2}} \Omega X^{\frac {1}{2}}$ is the vector from *X* to *Y* (Pennec et al. 2006), so $X^{-\frac {1}{2}} \Omega X^{-\frac {1}{2}}$ is essentially a measure of trajectory discrepancy *rescaled byX*.

As with the vector trajectory previously, we cannot say that a given interpolation will always the most accurate one. However, one of the error terms disappears for the AI geodesic interpolation; all other things being equal, it is reasonable to expect that the error on the geodesic interpolation will be, at the very least, less variable than the error on the entry-wise linear interpolation. For extrapolation, the error estimate is no longer relevant for the entry-wise linear method because such an extrapolation is not guaranteed to remain positive-semidefinite. However, the error on the AI geodesic extrapolation is well-defined by the remainder formula in Taylor’s theorem. For example, extrapolating past *R*_1_ to *t*>1 using an AI geodesic built by interpolating from *R*_0_ to *R*_1_ would produce the following error bound:

40 $$ d^{2} \left(X(t),Y(t)\right) \leq \left(t-1\right) \left| \frac{d}{dt} \left(d^{2} \left(X,Y\right)\right) \right|_{t=1} + \frac{1}{2} \left(t-1\right)^{2} \max \limits_{\tau \in \left[1,t\right]} \left| \frac{d^{2}}{dt^{2}} \left(d^{2}\left(X,Y\right) \right) \right|   $$

with the derivatives as previously calculated.

#### LE geodesic

For the LE geodesic,

41 $$\begin{array}{*{20}l} d^{2}\left(X,Y\right) &= \left\| \ln X - \ln Y \right\|^{2} = \text{tr} \left(\Omega^{2}\right) \end{array} $$

42 $$\begin{array}{*{20}l} \Omega &= V - W \end{array} $$

43 $$\begin{array}{*{20}l} V &= \ln X \end{array} $$

44 $$\begin{array}{*{20}l} W &= \ln Y \end{array} $$

45 $$\begin{array}{*{20}l} \frac{d}{dt} \left(d^{2}\left(X,Y\right)\right) &= 2 \text{tr} \left(\Omega \dot{\Omega}\right) \end{array} $$

46 $$\begin{array}{*{20}l} \text{tr} \left(\Omega \dot{\Omega}\right) &= \text{tr} \left(\left(V - W\right) \left(\dot{V} - \dot{W}\right) \right)  \\ &= \text{tr} \left(V \dot{V}\right) - \text{tr} \left(W \dot{V}\right) - \text{tr} \left(V \dot{W}\right) + \text{tr} \left(W \dot{W}\right) \end{array} $$

47 $$\begin{array}{*{20}l} \frac{d}{dt} \left(\text{tr} \left(\Omega \dot{\Omega}\right) \right) &= \text{tr} \left(\frac{d}{dt} \left(V \dot{V}\right)\right) - \text{tr} \left(\frac{d}{dt} \left(W \dot{V}\right)\right) - \text{tr} \left(\frac{d}{dt} \left(V \dot{W}\right)\right)\\ &\quad + \text{tr} \left(\frac{d}{dt} \left(W \dot{W}\right)\!\right) \end{array} $$

In general, all of these derivative terms will be non-zero. However, for the LE geodesic interpolation

48 $$\begin{array}{*{20}l} Y &= \exp \left(\left(1-t\right) \ln S_{2} + t \ln S_{1}\right) \end{array} $$

49 $$\begin{array}{*{20}l} W &= \ln Y = \left(1-t\right) \ln S_{2} + t \ln S_{1} \end{array} $$

50 $$\begin{array}{*{20}l} \dot{W} &= \frac{d}{dt} \left(\ln Y\right) = \ln S_{2} - \ln S_{1} \end{array} $$

51 $$\begin{array}{*{20}l} \ddot{W} &= 0 \end{array} $$

Several of the terms in $\frac {d}{dt} \left (\text {tr} \left (\Omega \dot {\Omega }\right)\right)$ will therefore be zero for the LE geodesic. As such, we would expect the error from the LE geodesic to be less than the error from the entry-wise input interpolation for the same reasons that we would expect the AI geodesic error to be smaller than the entry-wise linear interpolation. We can then plug these results into Eq. 40 to get error bounds for the LE geodesic.

## Additional files

Additional file 1 The synthetic data snapshot files begin with ‘comm-fixed’, and each snapshot file is suffixed with its time index. The proteomics snapshot data files begin with “ppn-numeric” and they are also suffixed with their time indices. The.edges files may be read with a text editor; we recommend TextPad. (ZIP 46 kb)

Additional file 2 The Python implementation of the methods described in the paper. (PY 24 kb)

## References

[CR1] Absil PA, Mahony R, Sepulchre R (2007). Optimization Algorithms on Matrix Manifolds.

[CR2] Arsigny V, Fillard P, Pennec X, Ayache N (2007). Geometric means in a novel vector space structure on symmetric positive-definite matrices. SIAM J Matrix Anal Appl.

[CR3] Batson J, Spielman DA, Srivastava N, Ten SH (2013). Spectral sparsification of graphs: Theory and algorithms. Commun ACM.

[CR4] Blondel, V, Guillaume JL, Lambiotte R, Lefebvre E (2008) Fast unfolding of communities in large networks. J Stat Mech Theory Exp. P10008. http://iopscience.iop.org/article/10.1088/1742-5468/2008/10/P10008/pdf.

[CR5] Boccaletti S, Latora V, Moreno Y, Chavez M, Hwang DU (2006). Compex networks, structure and dynamics. Phys Rep.

[CR6] Boguná M, Papadopoulos F, Krioukov D (2010). Sustaining the internet with hyperbolic mapping. Nat Commun.

[CR7] Bonnabel S, Sepulchre R (2009). Riemannian metric and geometric mean for positive semidefinite matrices of fixed rank. SIAM J Matrix Anal Appl.

[CR8] Boothby WM (1986). An Introduction to Differentiable Manifolds and Riemannian Geometry.

[CR9] Brualdi, RA (2006) Energy of a graph In: Notes to AIM Workshop on Spectra of Families of Matrices Described by Graphs, Digraphs, and Sign Patterns.

[CR10] Cazabet R, Amblard F, Alhajj R, Rokne J (2014). Dynamic Community Detection. Encyclopedia of Social Network Analysis and Mining.

[CR11] Domokos G, Sipos AR, Szabó T (2012). The mechanics of rocking stones:equilibria of separated scales. Math Geosci.

[CR12] Fenn DJ, Porter MA, Mucha PJ, McDonald M, Williams S, Johnson NF, Jones NS (2012). Dynamical clustering of exchange rates. Quant Finan.

[CR13] Fortunato S (2010). Community detection in graphs. Phys Rep.

[CR14] Girvan M, Newman ME (2002). Community structure in social and biological networks. Proc Natl Acad Sci.

[CR15] Greene J (2014). Traces of matrix products. Electron J Matrix Algebra..

[CR16] Harris JM, Hirst JL, Mossinghoff M (2008). Combinatorics and Graph Theory.

[CR17] Keshava Prasad T, Goel R, Kandasamy K, Keerthikumar S, Kumar S, Mathivanan S, Telikicherla D, Raju R, Shafreen B, Venugopal A (2008). Human protein reference database—2009 update. Nucleic acids Res.

[CR18] Krioukov D, Papadopoulos F, Vahdat A, Boguñá M (2009). Curvature and temperature of complex networks. Phys Rev E.

[CR19] Krioukov D, Papadopoulos F, Kitsak M, Vahdat A, Boguñá M (2010). Hyperbolic geometry of complex networks. Phys Rev E.

[CR20] Lambiotte R, Delvenne JC, Barahona M (2014). Random walks, markov processes and the multiscale modular organization of complex networks. IEEE Trans Netw Sci Eng.

[CR21] Mitchell HD, Eisfeld AJ, Sims AC, McDermott JE, Matzke MM, Webb-Robertson BJM, Tilton SC, Tchitchek N, Josset L, Li C (2013). A network integration approach to predict conserved regulators related to pathogenicity of influenza and sars-cov respiratory viruses. PLoS ONE.

[CR22] Mucha PJ, Richardson T, Macon K, Porter MA, Onnela JP (2010). Community structure in time-dependent, multiscale, and multiplex networks. Science.

[CR23] Newman MEJ (2010). Networks: An Introduction.

[CR24] Nguyen NP, Dinh TN, Shen Y, Thai MT (2014). Dynamic social community detection and its applications. Plos ONE.

[CR25] Pennec X, Fillard P, Ayache N (2006). A riemannian framework for tensor computing. Int J Comput Vis.

[CR26] Rand WM (1971). Objective criteria for the evaluation of clustering methods. J Am Stat Assoc.

[CR27] Tantipathananandh C, Berger-Wolf TY (2011). Finding communities in dynamic social networks. 2011 IEEE 11th International Conference on Data Mining.

[CR28] Traag VA, Bruggeman J (2009). Community detection in networks with positive and negative links. Phys Rev E.

[CR29] Vandereycken B (2013). Low-rank matrix completion by riemannian optimization. SIAM J Optim.

